# Supplementary material for: Spatio-temporal transcriptomics of chromothriptic SHH-medulloblastoma identifies multiple genetic clones that resist treatment and drive relapse
Source: Nat Commun. 2024 Nov 29;15:10370. doi: 10.1038/s41467-024-54709-w (PMC11604656; doi:10.1038/s41467-024-54709-w)
Supplement: Supplementary file 1 — Supplementary Information [file 41467_2024_54709_MOESM1_ESM.pdf]

Spatiotemporal transcriptomics of chromothriptic SHH-medulloblastoma identifies multiple genetic clones that resist treatment and drive relapse

Supplementary Figures

Supplementary Figure 1

A

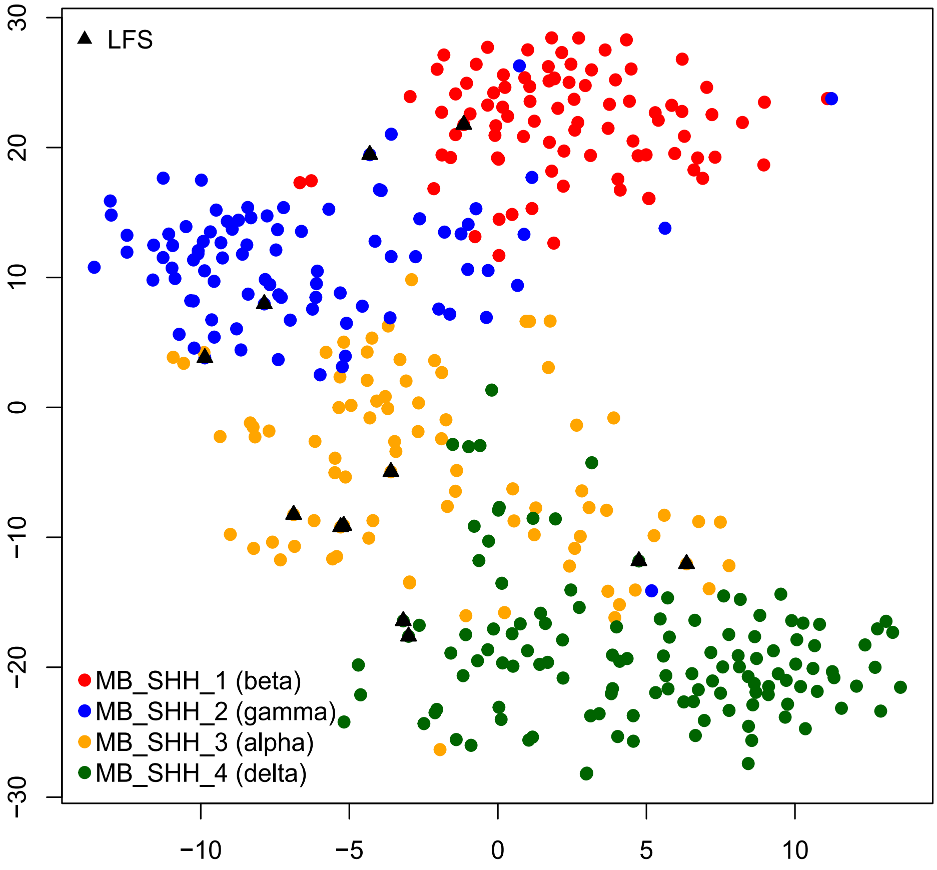

B

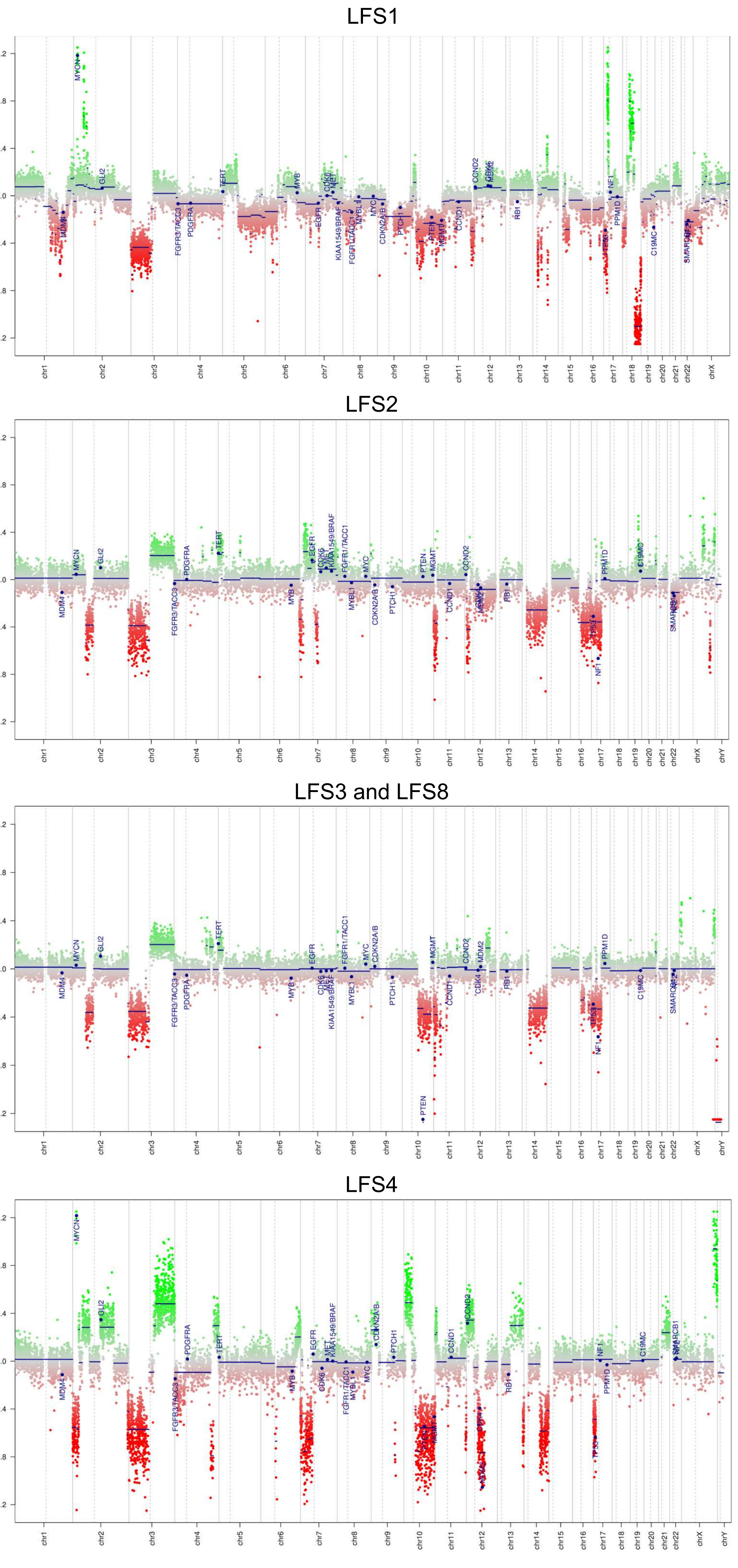
LFS1
LFS2
LFS3 and LFS8
LFS4
1.2
0.8
0.4
0.0
−0.4
−0.8
−1.2
chr1
chr2
chr3
chr4
chr5
chr6
chr7
chr8
chr9
chr10
chr11
chr12
chr13
chr14
chr15
chr16
chr17
chr18
chr19
chr20
chr21
chr22
chrX
chrY

LFS5

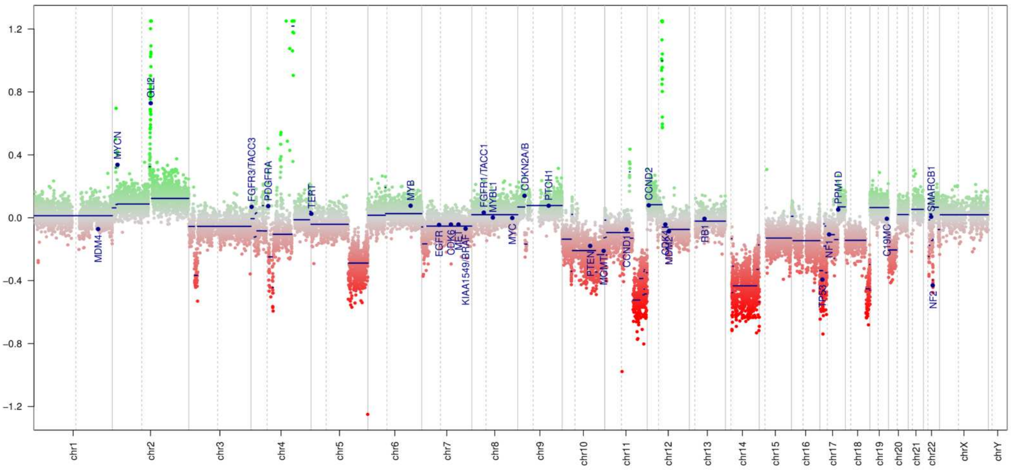

LFS6 and LFS7

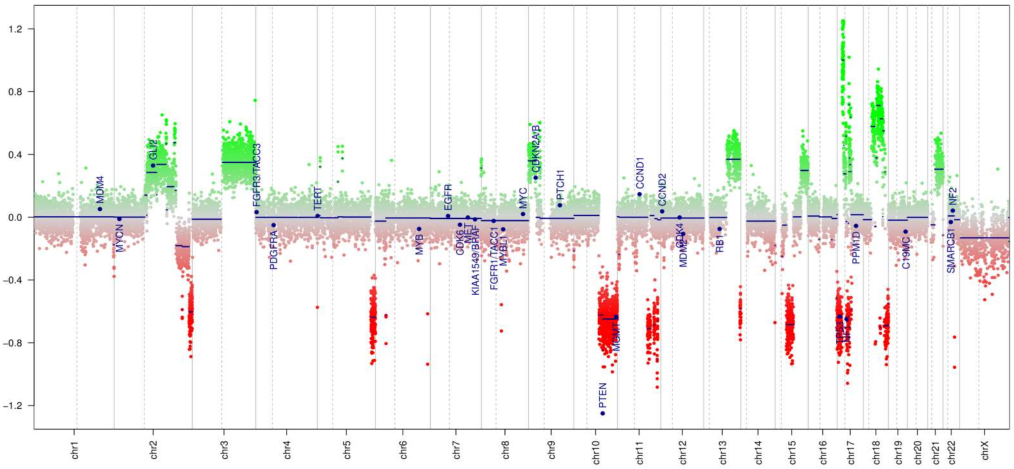

S1

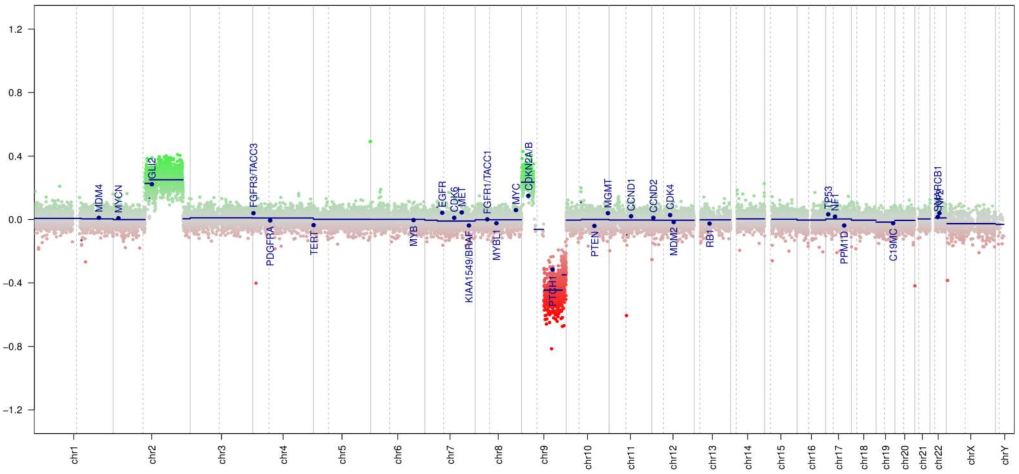

S2

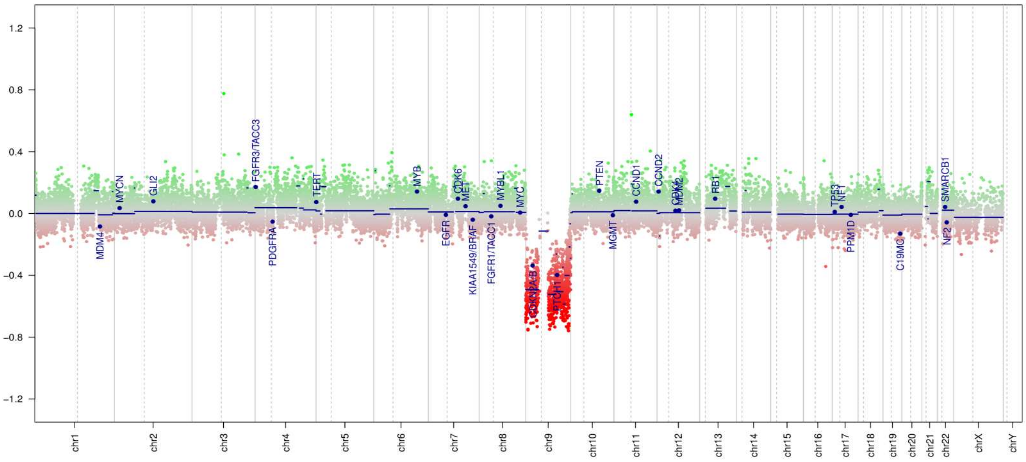
1.2
0.8
0.4
0.0
−0.4
−0.8
−1.2
MDM4
MYCN
GLI2
FGFR3/TACC3
PDGFRA
TERT
MYB
EGFR
CDK6
MET
KIAA1549/BRAF
FGFR1/TACC1
MYBL1
MYC
PTEN
MGMT
CCND1
CCND2
CDK4
MDM2
RB1
TP53
NF1
PPM1D
C19MC
SMARCB1
NF2
chr1
chr2
chr3
chr4
chr5
chr6
chr7
chr8
chr9
chr10
chr11
chr12
chr13
chr14
chr15
chr16
chr17
chr18
chr19
chr20
chr21
chr22
chrX
chrY

S3

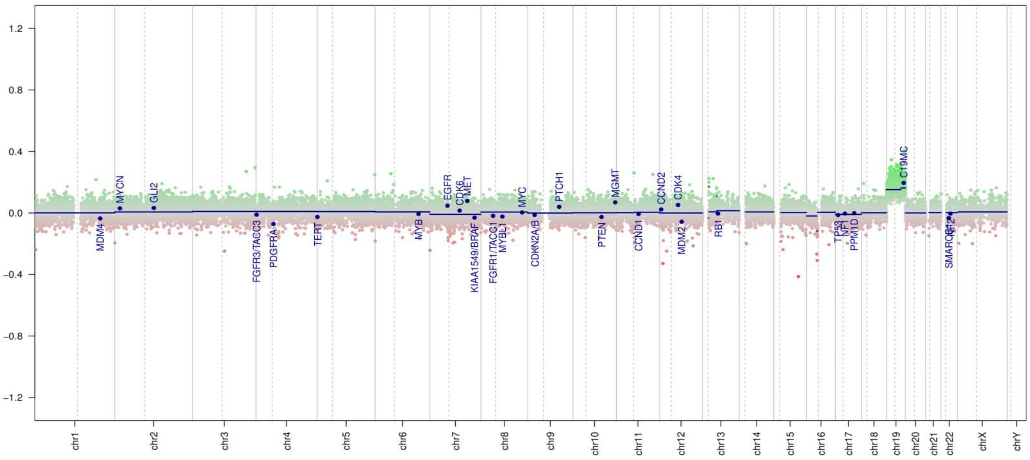
1.2
0.8
0.4
0.0
−0.4
−0.8
−1.2
MDM4
MYCN
GLI2
FGFR3/TACC3
PDGFRA
TERT
MYB
EGFR
CDK6
MET
KIAA1549/BRAF
FGFR1/TACC1
MYBL1
MYC
CDKN2A/B
PTCH1
PTEN
MGMT
CCND1
CCND2
CDK4
MDM2
RB1
TP53
NF1
PPM1D
C19MC
SMARCB1
NF2
chr1
chr2
chr3
chr4
chr5
chr6
chr7
chr8
chr9
chr10
chr11
chr12
chr13
chr14
chr15
chr16
chr17
chr18
chr19
chr20
chr21
chr22
chrX
chrY

S4

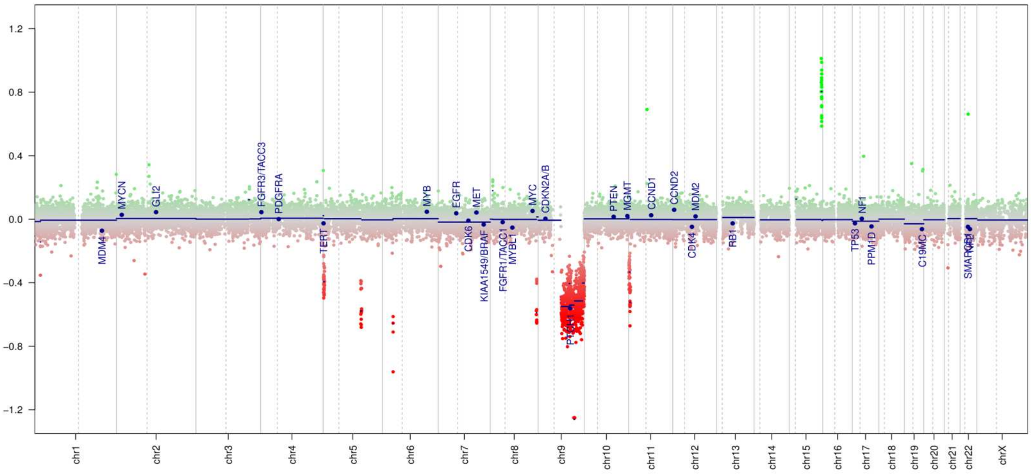
1.2
0.8
0.4
0.0
−0.4
−0.8
−1.2
MDM4
MYCN
GLI2
FGFR3/TACC3
PDGFRA
TERT
MYB
EGFR
CDK6
MET
KIAA1549/BRAF
FGFR1/TACC1
MYBL1
MYC
CDKN2A/B
PTCH1
PTEN
MGMT
CCND1
CCND2
CDK4
MDM2
RB1
TP53
NF1
PPM1D
C19MC
SMARCB1
chr1
chr2
chr3
chr4
chr5
chr6
chr7
chr8
chr9
chr10
chr11
chr12
chr13
chr14
chr15
chr16
chr17
chr18
chr19
chr20
chr21
chr22
chrX

S5

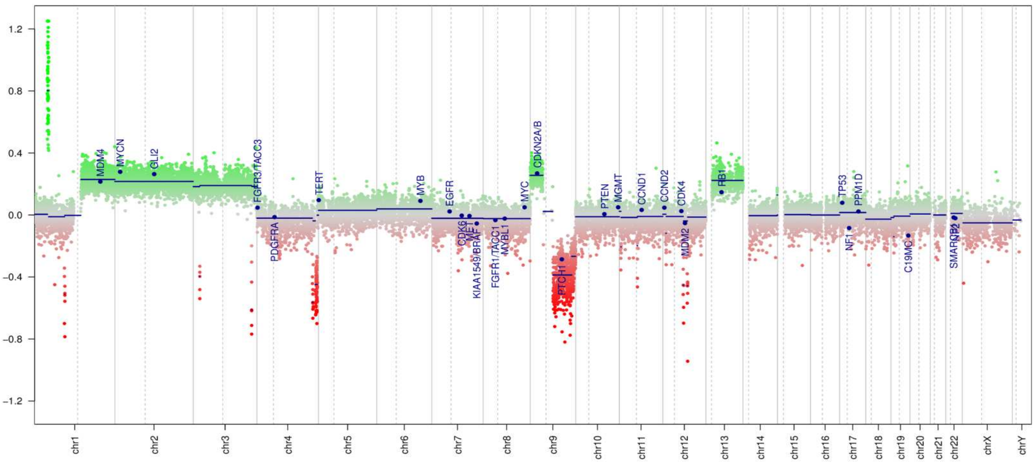
1.2
0.8
0.4
0.0
−0.4
−0.8
−1.2
MDM4
MYCN
GLI2
FGFR3/TACC3
PDGFRA
TERT
MYB
EGFR
CDK6
MET
KIAA1549/BRAF
FGFR1/TACC1
MYBL1
MYC
CDKN2A/B
PTCH1
PTEN
MGMT
CCND1
CCND2
CDK4
MDM2
RB1
TP53
NF1
PPM1D
C19MC
SMARCB1
NF2
chr1
chr2
chr3
chr4
chr5
chr6
chr7
chr8
chr9
chr10
chr11
chr12
chr13
chr14
chr15
chr16
chr17
chr18
chr19
chr20
chr21
chr22
chrX
chrY

Supplementary Figure 1.

A. t-SNE showing a clustering of the medulloblastoma cohort (n=13, black triangles) with a reference cohort of molecularly characterised medulloblastomas (circles). All samples belong to the SHH molecular subgroup of medulloblastoma.

B. Copy-number plots from methylation arrays for all medulloblastomas. LFS3 and LFS8, as well as LFS6 and LFS7, respectively, are two Visium samples from different tumour regions from the same patients.

Supplementary Figure 2

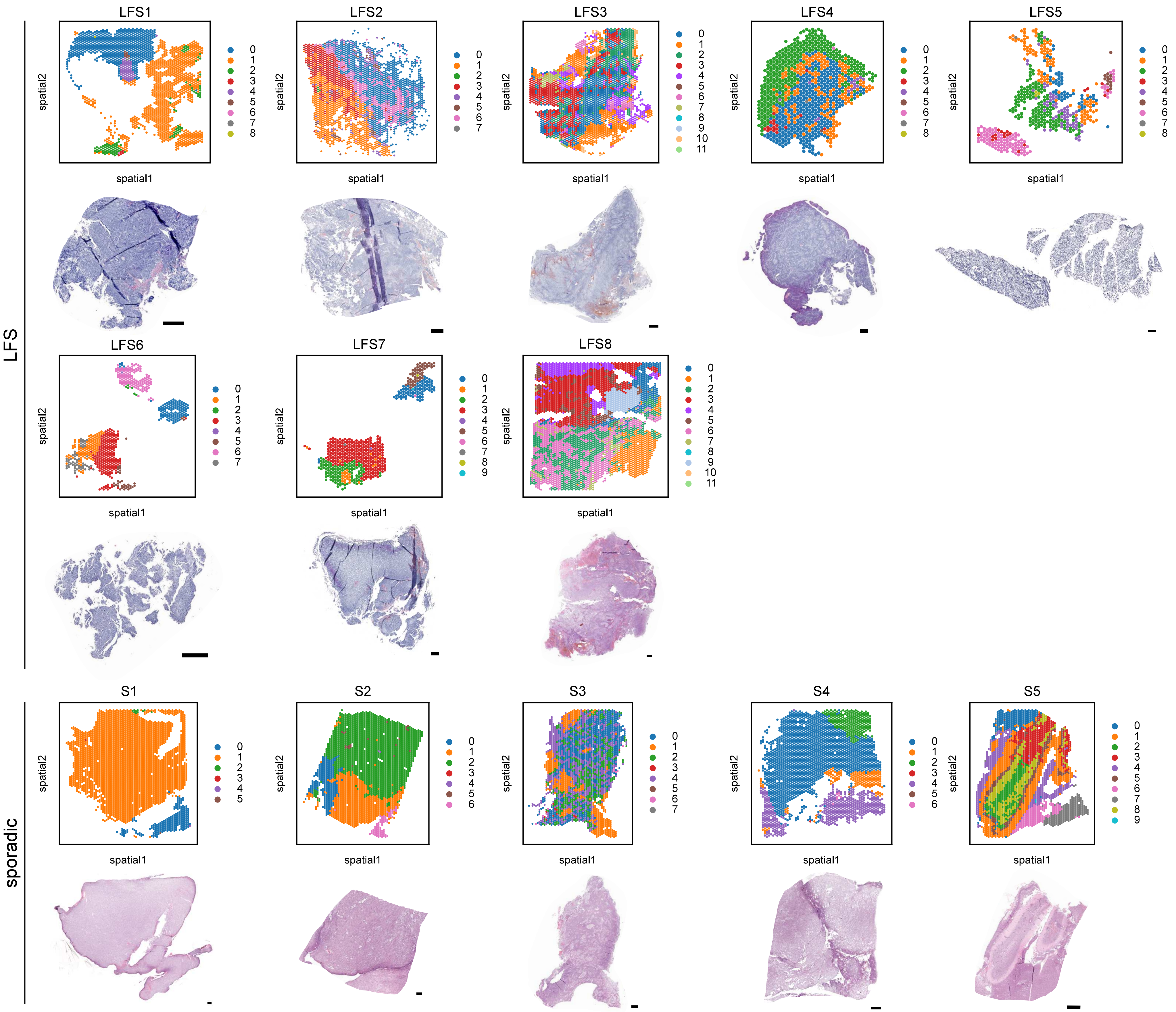

Supplementary Figure 2. Spatial gene expression shows more intra-tumour transcriptional heterogeneity in LFS medulloblastoma as compared to sporadic medulloblastoma. Clustering of human medulloblastoma tissue Visium data with SpatialDE2 segments the tissue into transcriptionally similar regions. Shown are all 13 samples. 1590 spots (LFS1), 2642 spots (LFS2), 2469 spots (LFS3), 881 spots (LFS4), 500 spots (LFS5), 631 spots (LFS6), 537 spots (LFS7), 3983 spots (LFS8), 2966 spots (S1), 2621 spots (S2), 2251 spots (S3), 2198 spots (S4), 2723 spots (S5). Hematoxylin and eosin stains of consecutive sections of all human medulloblastomas used for Visium analysis.

Supplementary Figure 3

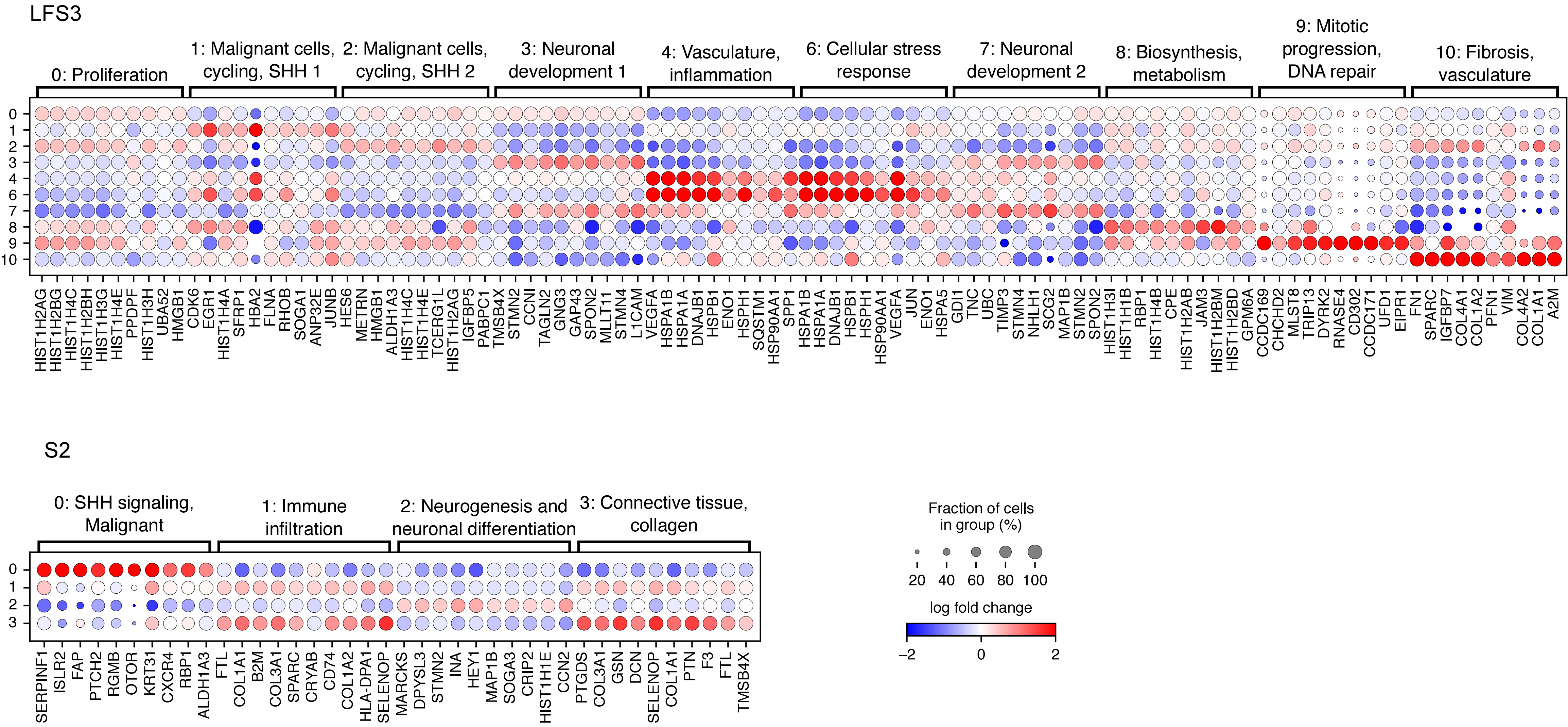

Supplementary Figure 3. Dot plot showing top differentially expressed genes between tissue regions for medulloblastomas shown in Figure 1.

Supplementary Figure 4

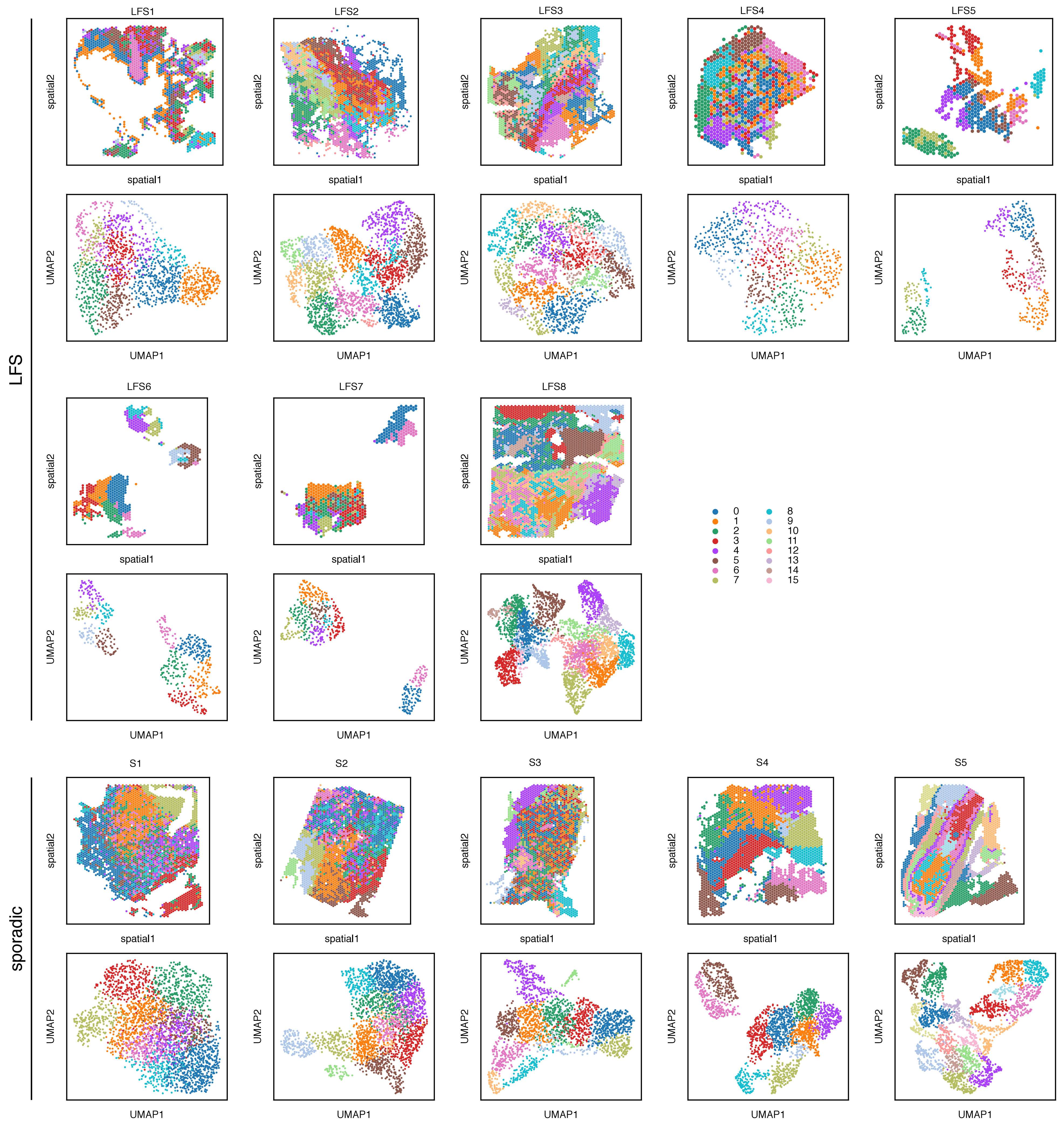

Supplementary Figure 4. Leiden clustering for LFS and sporadic medulloblastomas. Shown are all 13 samples. 1590 spots (LFS1), 2642 spots (LFS2), 2469 spots (LFS3), 881 spots (LFS4), 500 spots (LFS5), 631 spots (LFS6), 537 spots (LFS7), 3983 spots (LFS8), 2966 spots (S1), 2621 spots (S2), 2251 spots (S3), 2198 spots (S4), 2723 spots (S5).

Supplementary Figure 5

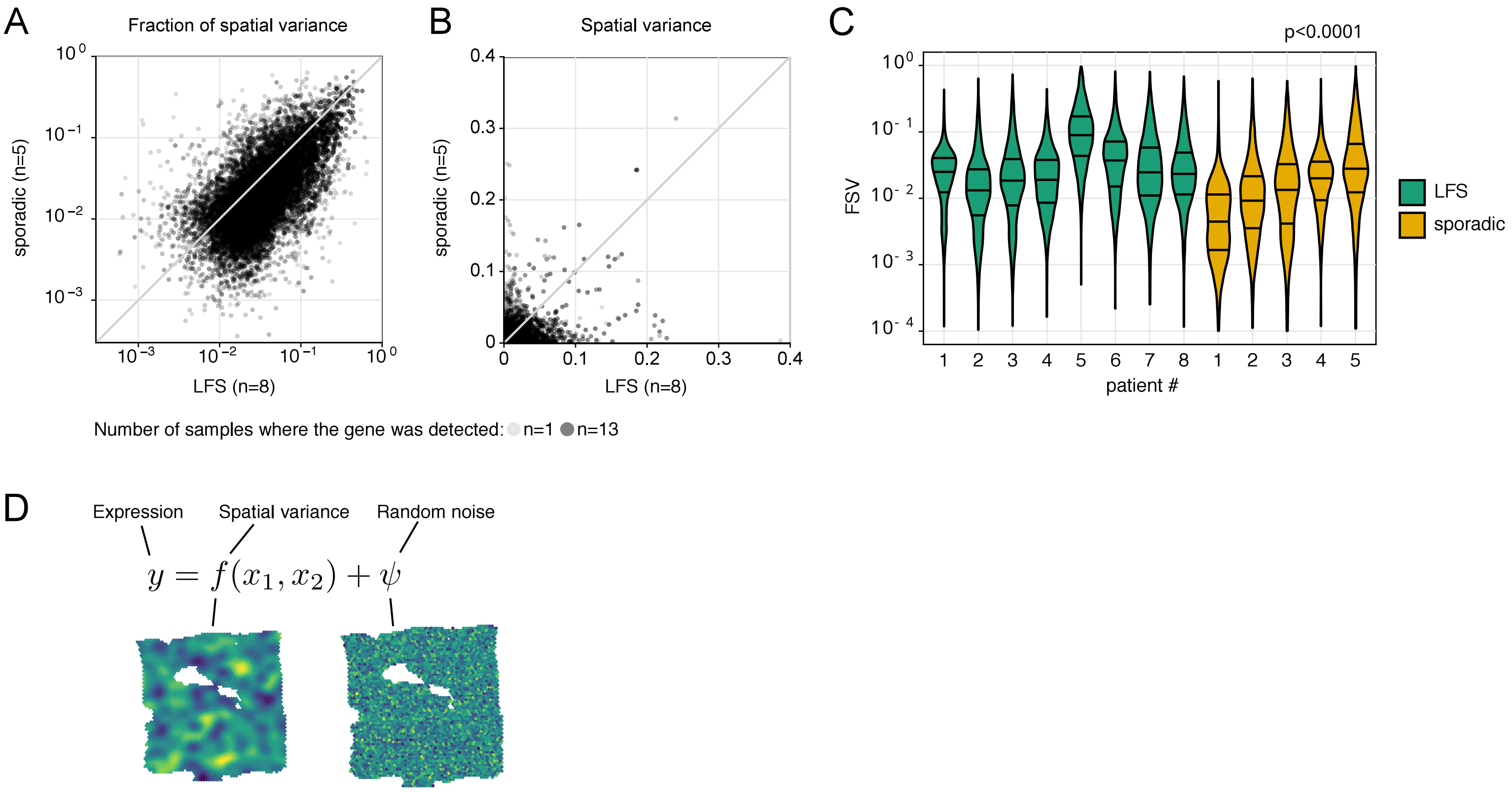

Supplementary Figure 5.

A. Scatter plot of the fraction of expression variance attributable to spatial location for LFS (x-axis) versus sporadic medulloblastomas (y-axis). Each dot corresponds to one gene; spatial variance estimates are averaged across samples.

B. Total variance attributed to spatial variability of gene expression by SpatialDE2 in LFS and sporadic medulloblastomas.

C. Distribution of the fraction of expression variance explained by spatial locations (FSV) for individual genes. LFS medulloblastomas show a significantly higher FSV as compared to sporadic tumours (Two sided Mann-Whitney U-test, n=12217 (LFS), n=12380 (sporadic)).

D. Cartoon representation of the strategy to evaluate spatial variance.

Supplementary Figure 6

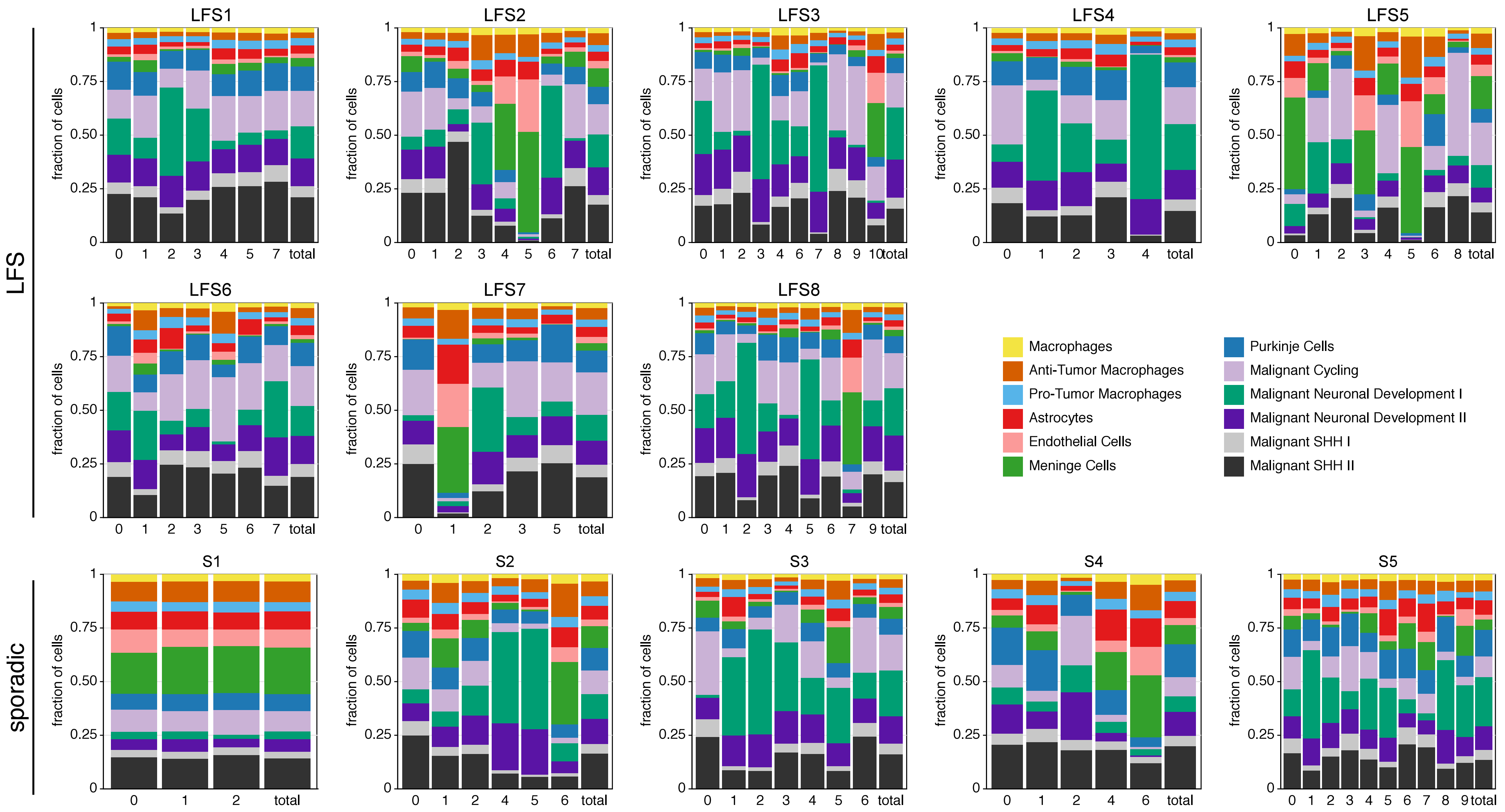

Supplementary Figure 6. Bar graphs showing the cell type abundance for each region in the human medulloblastomas.

Supplementary Figure 7

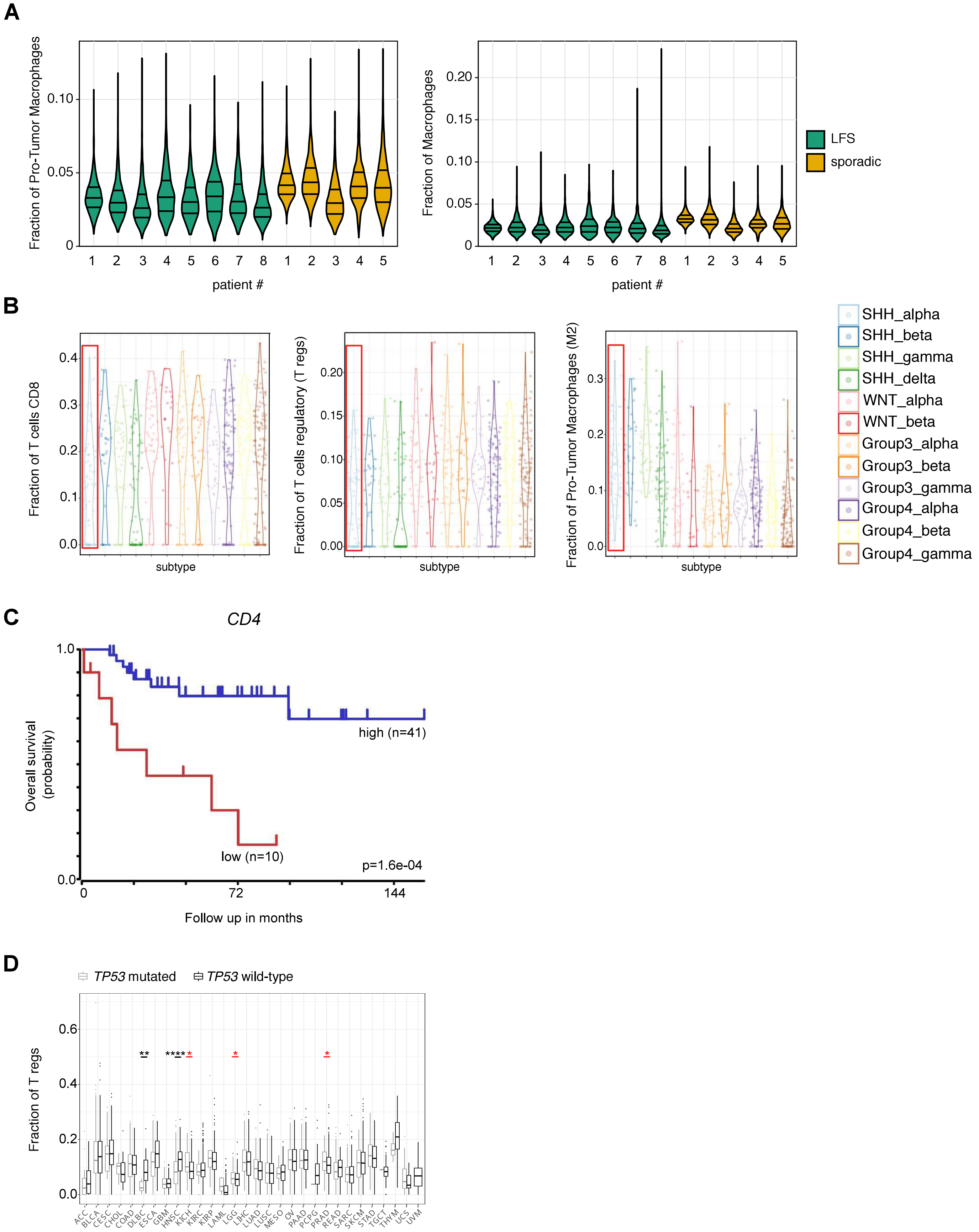

Supplementary Figure 7.

A. Fraction of pro-tumour macrophages and unpolarized macrophages in LFS (n=8) and sporadic (n=5) medulloblastomas in the Visium data (n=1590 (LFS1), n=2642 (LFS2), n=2469 (LFS3), n=881 (LFS4), n=500 (LFS5), n=631 (LFS6), n=537 (LFS7), n=3983 (LFS8), n=2966 (S1), n=2621 (S2), n=2251 (S3), n=2198 (S4), n=2723 (S5)).

B. Less T-cells but more pro-tumour macrophages in SHH-alpha medulloblastoma (enriched for LFS medulloblastomas) as compared to other molecular subtypes (re-analysis of bulk RNA-seq data from Cavalli et al[1], n=763 medulloblastomas).

C. Low expression of *CD4* is linked with poor outcome in SHH medulloblastoma (n=51, log-rank test, Kaplan-Meier plots generated using the R2 database with the medulloblastoma cohort from Cavalli et al[1]).

D. Less T regs in *TP53* mutant DLBC and HNSC tumour entities (re-analysis of a pan-cancer cohort, deconvolution from bulk RNAseq, n=8,955[2]). Two-sided Mann-Whitney U-test was used. Boxes show the interquartile range (IQR), the line indicates the median, whiskers indicate 1.5x IQR.

Supplementary Figure 8

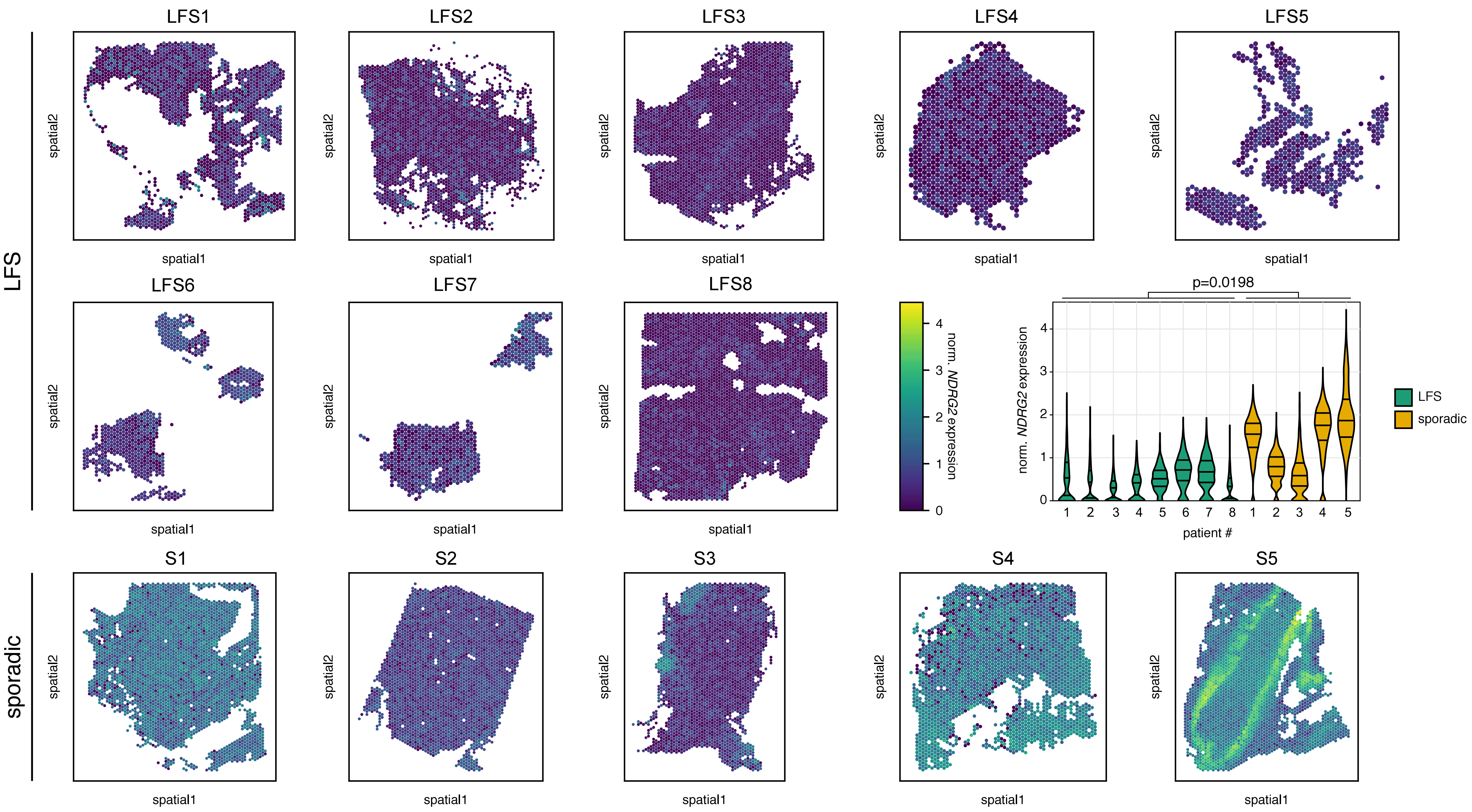

Supplementary Figure 8. Expression of tumour suppressor gene *NDRG2* in LFS and sporadic medulloblastoma. DESeq2 with IHW multiple testing correction. Shown are all 13 samples. 1590 spots (LFS1), 2642 spots (LFS2), 2469 spots (LFS3), 881 spots (LFS4), 500 spots (LFS5), 631 spots (LFS6), 537 spots (LFS7), 3983 spots (LFS8), 2966 spots (S1), 2621 spots (S2), 2251 spots (S3), 2198 spots (S4), 2723 spots (S5).

Supplementary Figure 9

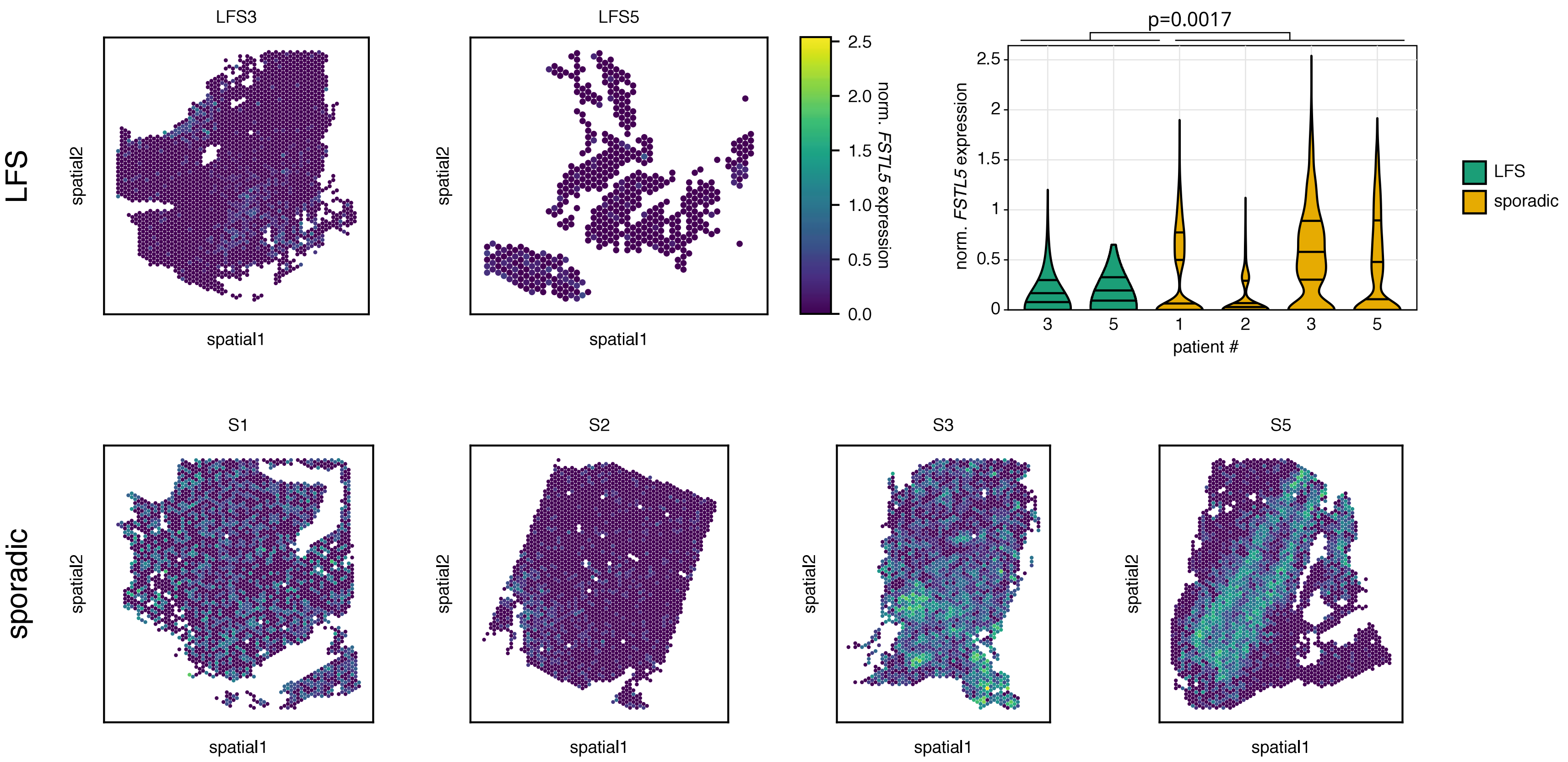

Supplementary Figure 9. Expression of tumour suppressor gene *FSTL5* in LFS and sporadic medulloblastoma. p-value: DESeq2 with IHW multiple testing correction (n=8 (LFS), n=5 (sporadic). In samples that are not shown the gene was removed during quality control due to low expression. 2469 spots (LFS3), 500 spots (LFS5), 2966 spots (S1), 2621 spots (S2), 2251 spots (S3), 2723 spots (S5).

Supplementary Figure 10

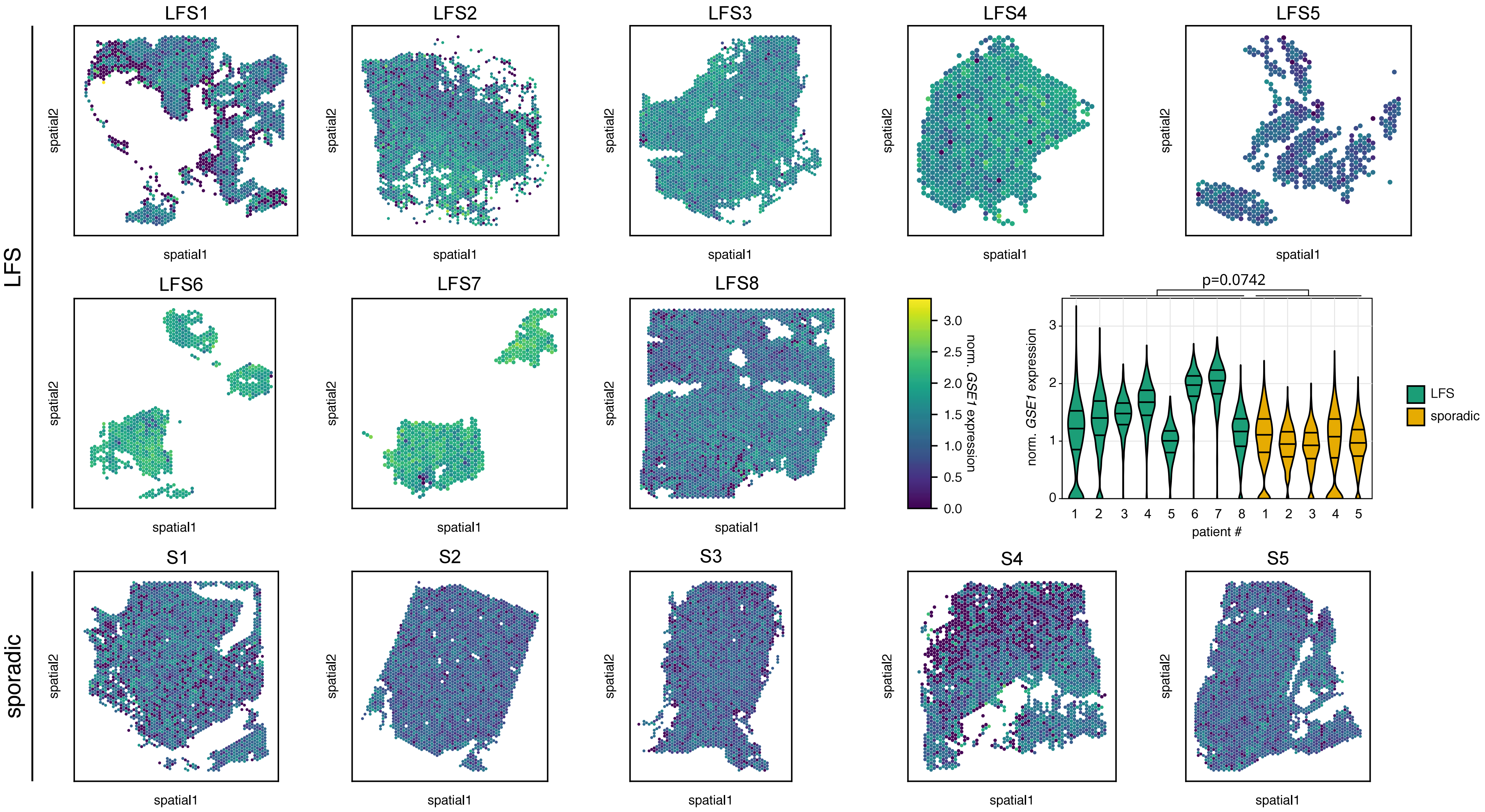

Supplementary Figure 10. Expression of oncogene *GSE1* in LFS and sporadic medulloblastoma. Statistical analysis was performed using DESeq2 with IHW multiple testing correction (n=8 (LFS), n=5 (sporadic)). 1590 spots (LFS1), 2642 spots (LFS2), 2469 spots (LFS3), 881 spots (LFS4), 500 spots (LFS5), 631 spots (LFS6), 537 spots (LFS7), 3983 spots (LFS8), 2966 spots (S1), 2621 spots (S2), 2251 spots (S3), 2198 spots (S4), 2723 spots (S5).

Supplementary Figure 11

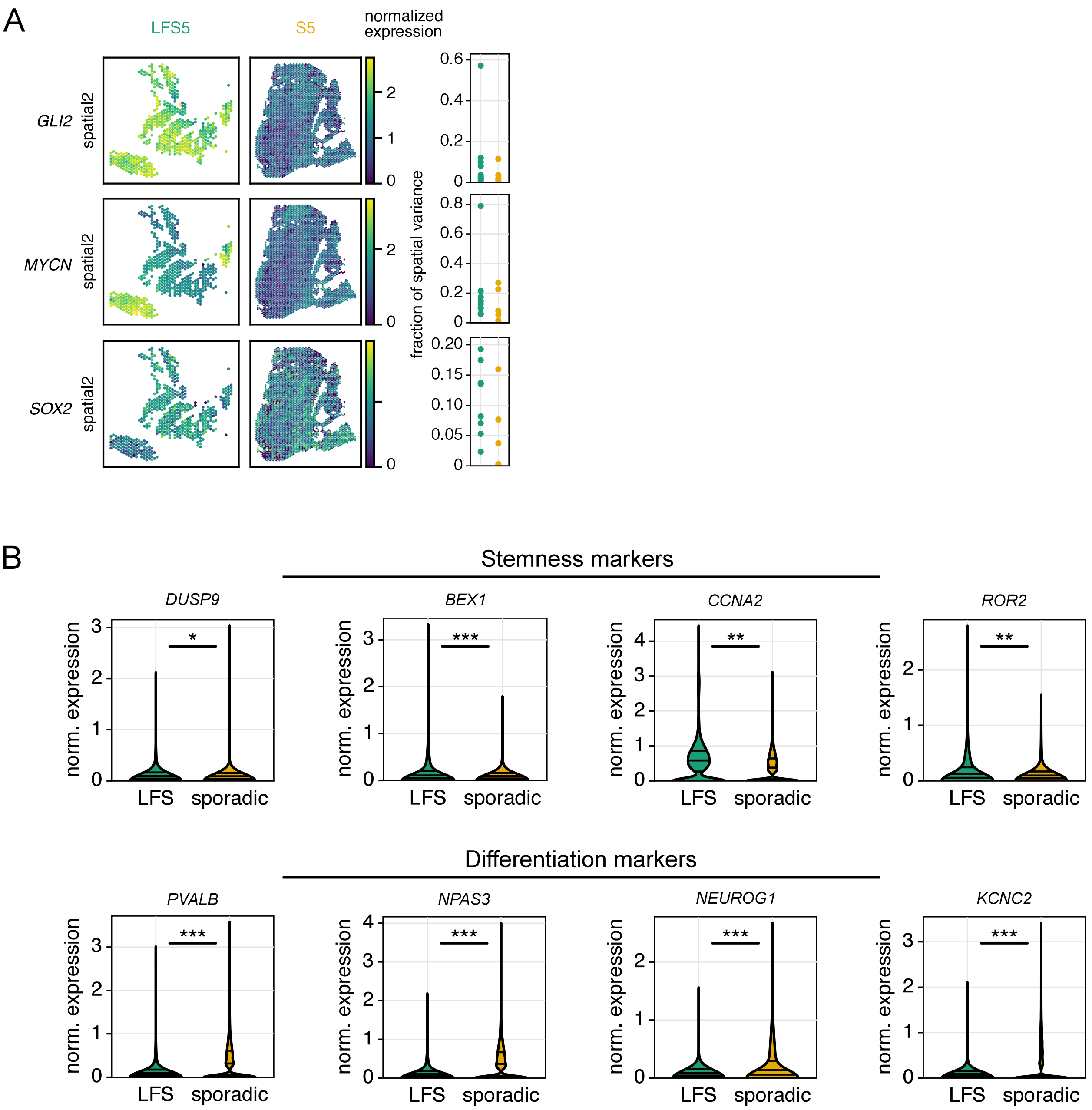

Supplementary Figure 11.

A. Spatial distribution of cancer drivers and stemness markers. Heterogeneous expression of oncogenes in LFS medulloblastoma (500 spots) compared to sporadic medulloblastoma (2723 spots). Left, representative examples; right, distribution of the fraction of spatial variance for the corresponding genes in LFS (n=8) and sporadic (n=5) tumours. Not significant, Mann-Whitney U-test.

B. Expression of stemness and differentiation markers in LFS and sporadic medulloblastomas. Statistical analysis was performed using DESeq2 with IHW multiple testing correction (*$p<0.1$, **$p<0.05$, ***$p<0.01$, n=8 (LFS), n=5 (sporadic)).

Supplementary Figure 12

A

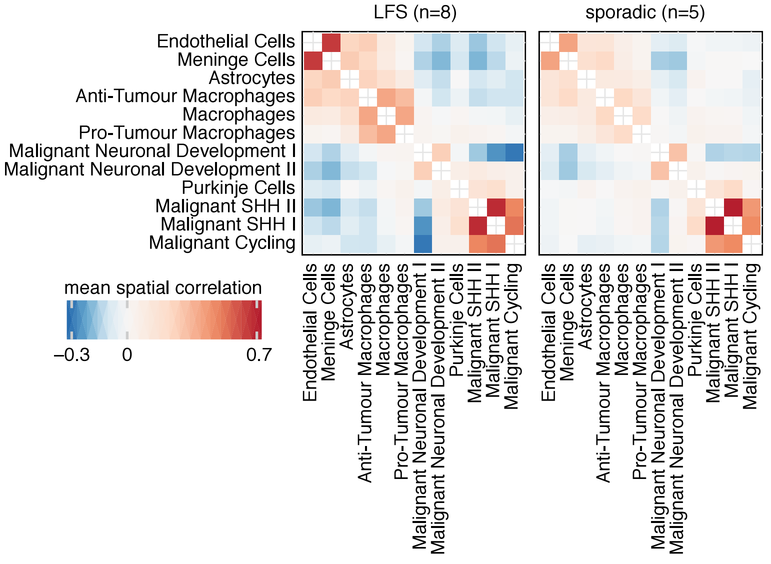

B

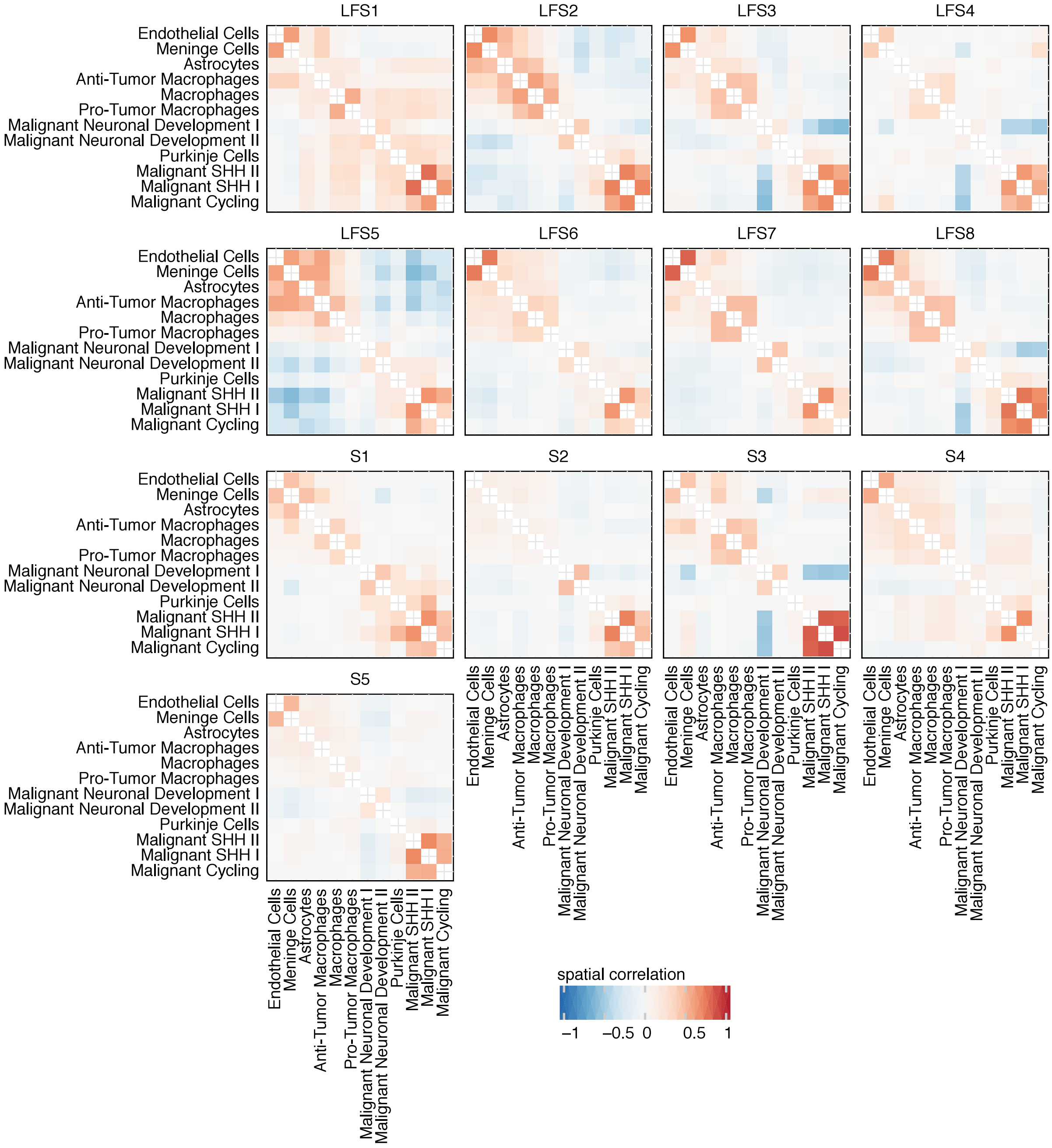

Supplementary Figure 12. Correlations of cell type co-occurrence at spatial locations.

A. Average correlation coefficients across LFS and sporadic tumours.

B. Correlation coefficients for individual samples.

Supplementary Figure 13

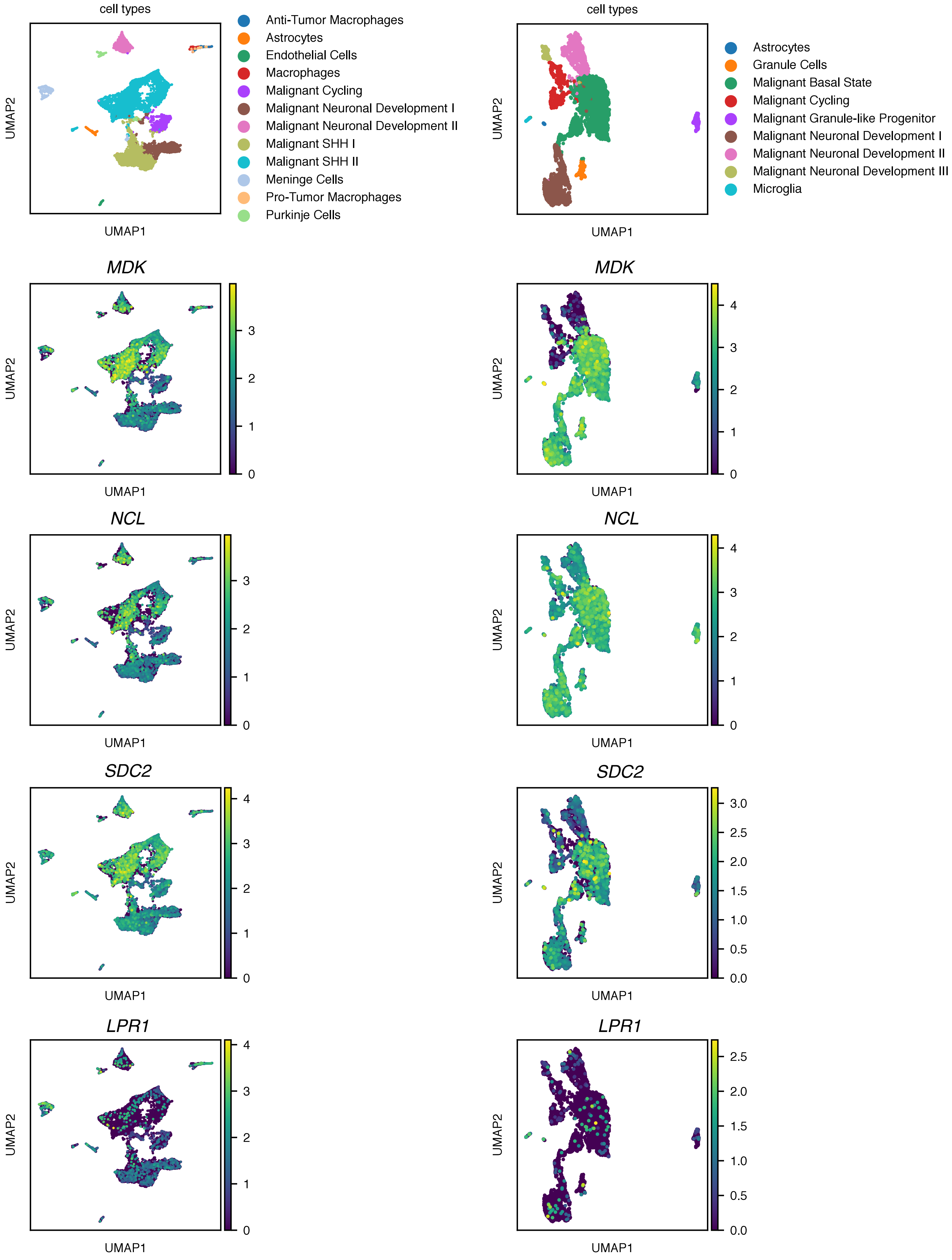

Supplementary Figure 13. UMAPs based on matched 10xRNAseq data for nuclei (LFS medulloblastoma patients, left side, 15265 cells from 3 samples) and PDX (right side, 7226 cells from 4 samples) with cell type annotation and expression of the *MDK* ligand as well as major receptors for midkine signalling in LFS medulloblastoma.

Supplementary Figure 14

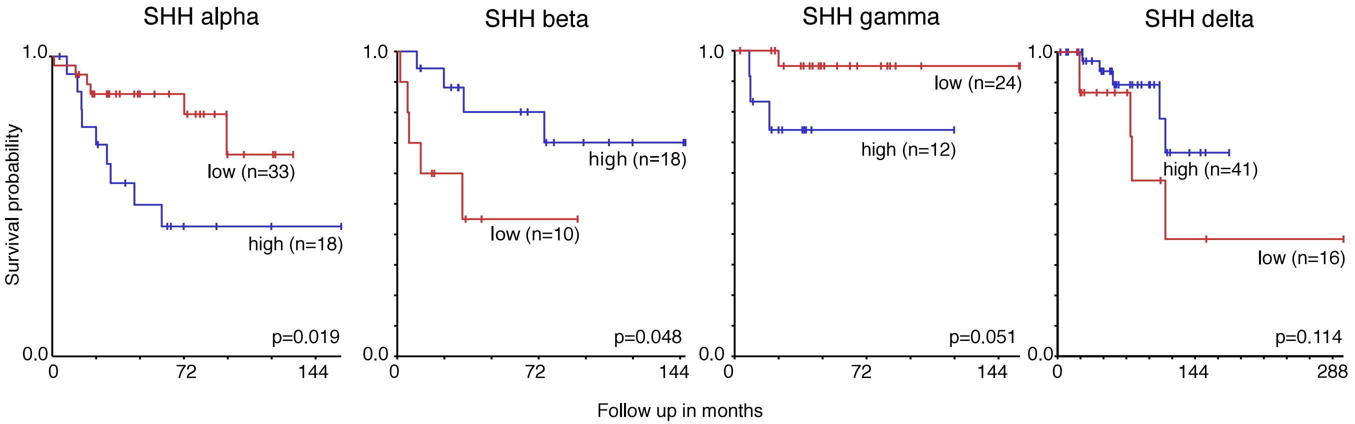

Supplementary Figure 14. Kaplan-Meier survival analysis performed using the R2 database (Tumour Medulloblastoma - Cavalli - 763 - rma_sketch - hugene11t dataset). High expression of *MDK* is associated with poor outcome in the SHH alpha subtype enriched for LFS medulloblastomas (log-rank test).

Supplementary Figure 15

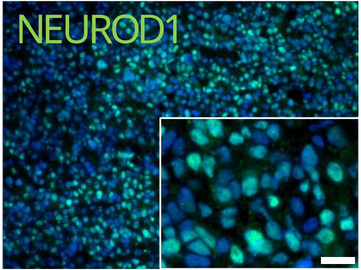

Supplementary Figure 15. Validation of NEUROD1 expression by immunofluorescence on tissue sections. The image is representative of one sample. Scale bar, 20 μm.

Supplementary Figure 16

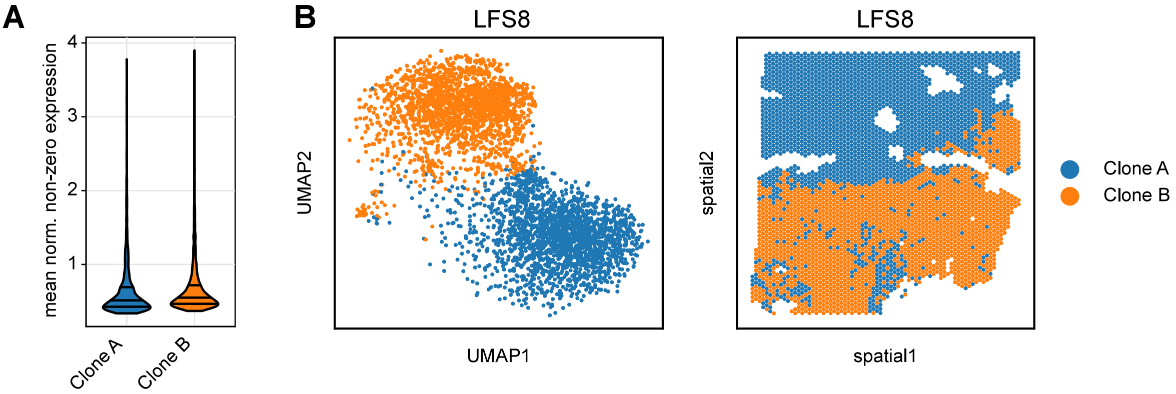

Supplementary Figure 16. Clone mapping strategy.

A. Mean expression of genes on chromosomes 8 and 12 for the two clones in human sample LFS8. Visium spots where a gene was not detected were excluded from the gene's mean calculation.

B. Leiden clustering of human sample LFS8 using only genes on chromosomes 8 and 12 (3983 spots).

Supplementary Figure 17

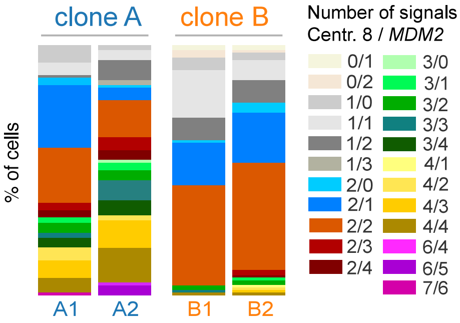

Supplementary Figure 17. FISH validation on consecutive tissue sections using probes for centromere 8 and *MDM2*, located in two chromosome regions that show different copy-numbers between the major genetic clones A and B. Quantification of FISH signals of each probe in areas A1, A2, B1 and B2 from the major clones A and B.

Supplementary Figure 18

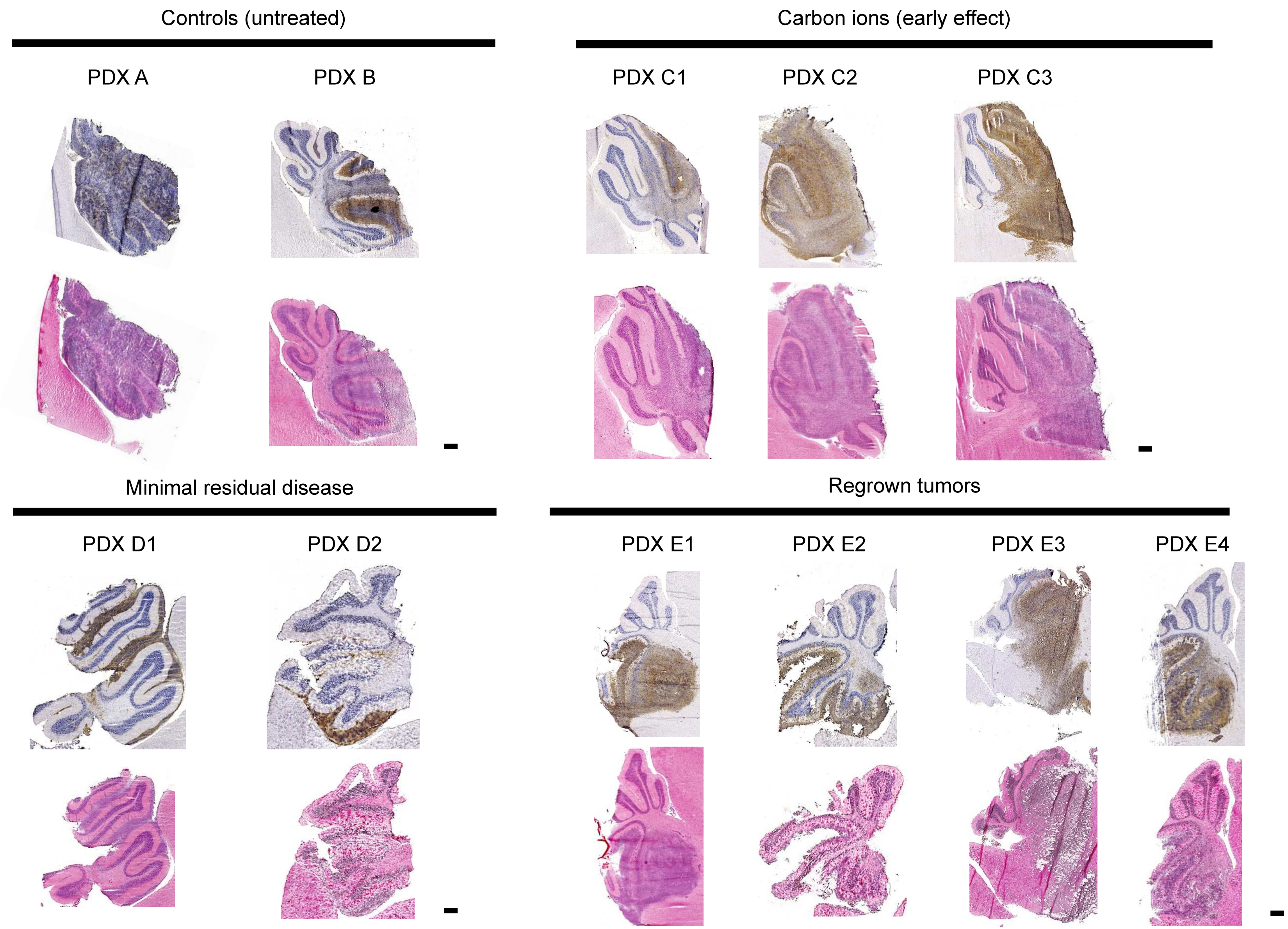

Supplementary Figure 18 STEM121 and haematoxylin and eosin stains for all 11 patient-derived xenografts used for Visium experiments. Scale bar, 500 μm.

Supplementary Figure 19

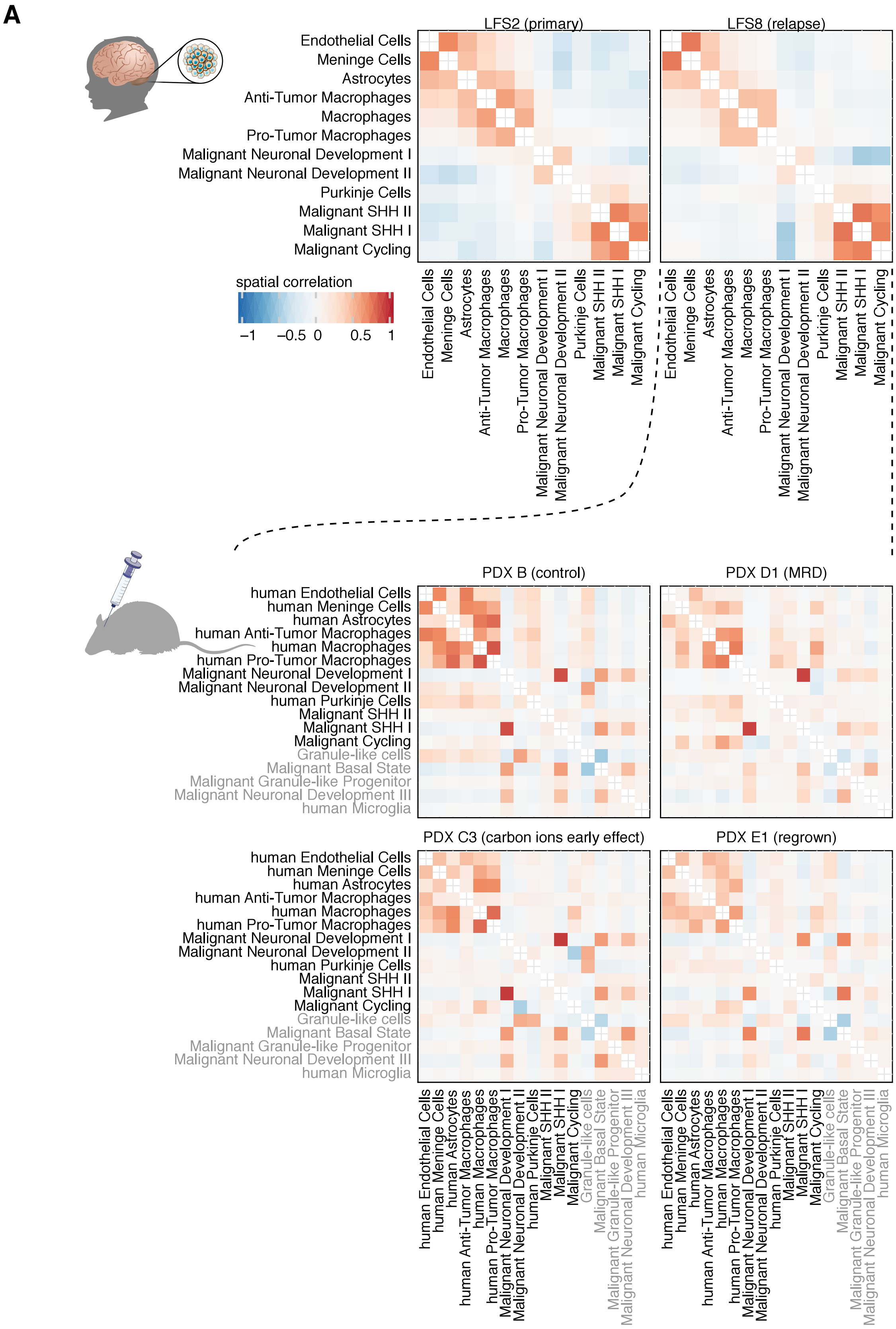

B

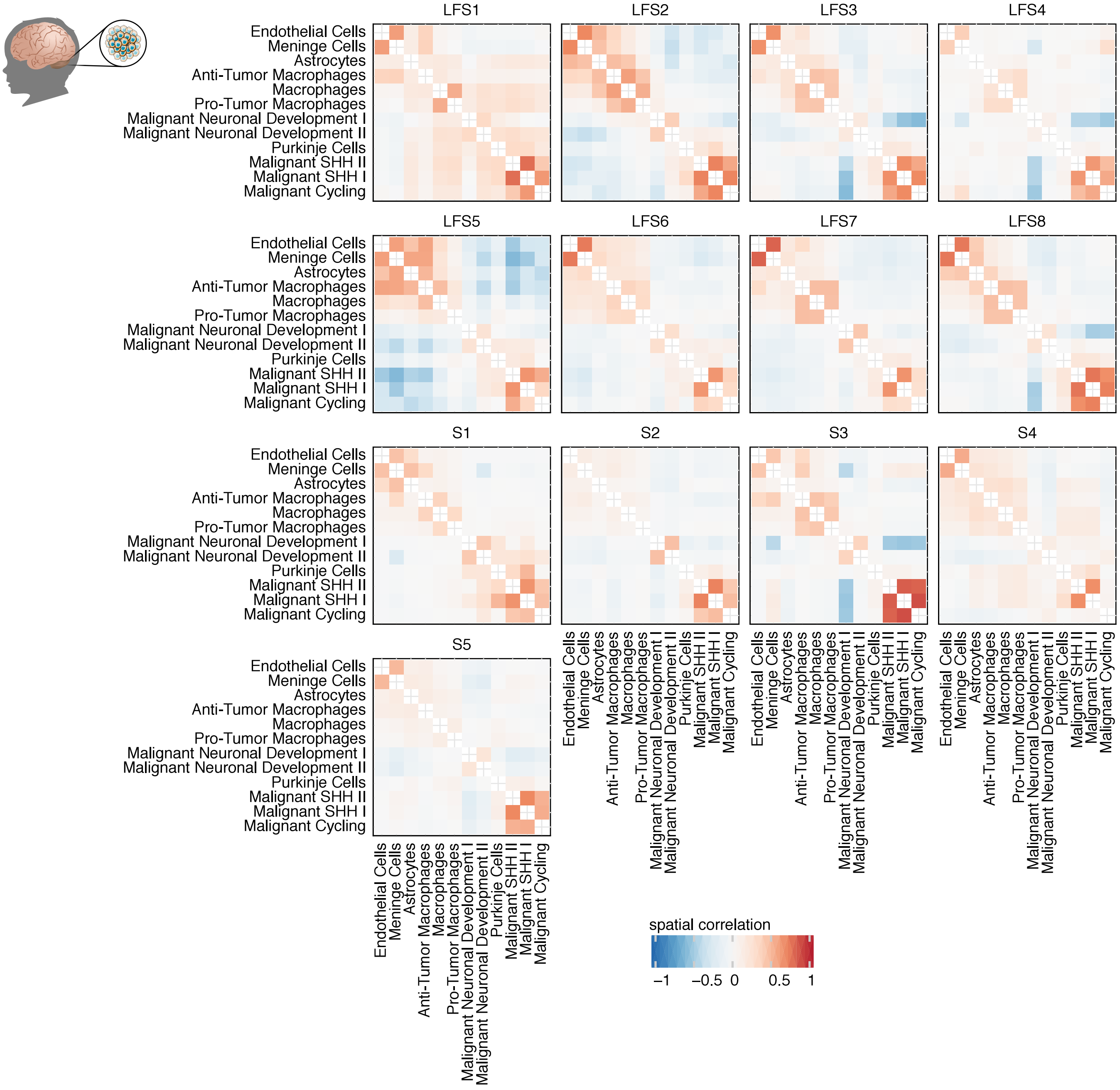
LFS1
LFS2
LFS3
LFS4
LFS5
LFS6
LFS7
LFS8
S1
S2
S3
S4
S5
Endothelial Cells
Meninge Cells
Astrocytes
Anti-Tumor Macrophages
Macrophages
Pro-Tumor Macrophages
Malignant Neuronal Development I
Malignant Neuronal Development II
Purkinje Cells
Malignant SHH II
Malignant SHH I
Malignant Cycling
spatial correlation
−1
−0.5
0
0.5
1

C

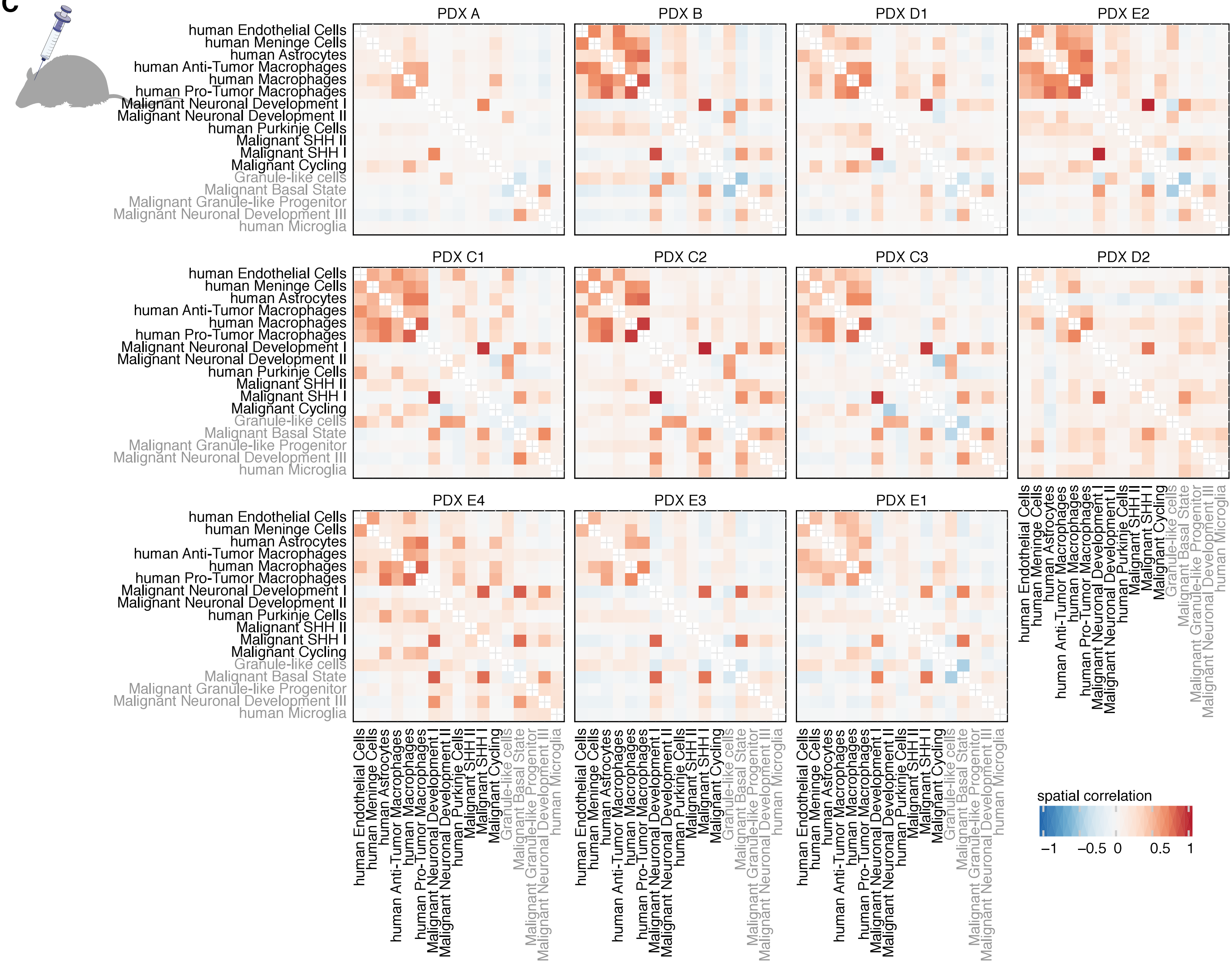
PDX A
PDX B
PDX D1
PDX E2
PDX C1
PDX C2
PDX C3
PDX D2
PDX E4
PDX E3
PDX E1
human Endothelial Cells
human Meninge Cells
human Astrocytes
human Anti-Tumor Macrophages
human Macrophages
human Pro-Tumor Macrophages
Malignant Neuronal Development I
Malignant Neuronal Development II
human Purkinje Cells
Malignant SHH II
Malignant SHH I
Malignant Cycling
Granule-like cells
Malignant Basal State
Malignant Granule-like Progenitor
Malignant Neuronal Development III
human Microglia
spatial correlation
−1
−0.5
0
0.5
1

D

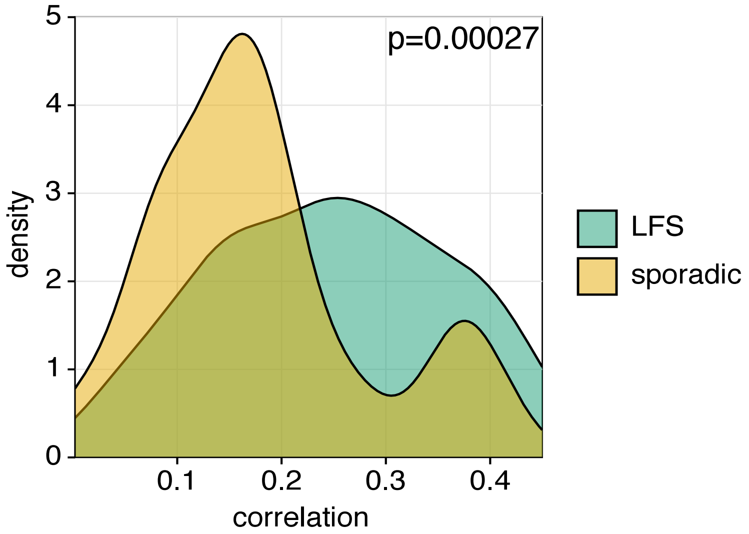
p=0.00027
density
correlation
0
1
2
3
4
5
0.1
0.2
0.3
0.4
LFS
sporadic

Supplementary Figure 19.

A. Spatial co-occurrence of cell-types in space. 2-D neighbourhood structure from the patient tumour is preserved in PDX and stable upon treatment. Cell types analysed only in PDX are indicated in grey. Head and brain icons are adapted from the Reactome icon library, used under CC BY 4.0 (https://creativecommons.org/licenses/by/4.0/). Rat, syringe, and cancerous cell icons are adapted from Bioicons (user Servier), used under CC BY 3.0 (https://creativecommons.org/licenses/by/3.0/).

B. Correlations of cell type abundance in space for all patients. Head and brain icons are adapted from the Reactome icon library, used under CC BY 4.0 (https://creativecommons.org/licenses/by/4.0/). Cancerous cell icon is adapted from Bioicons (user Servier), used under CC BY 3.0 (https://creativecommons.org/licenses/by/3.0/)

C. Correlations of cell type abundance in space for all patient-derived xenografts. Rat and syringe icons are adapted from Bioicons (user Servier), used under CC BY 3.0 (https://creativecommons.org/licenses/by/3.0/)

D. Distribution of cell type correlations between PDX and LFS or sporadic patient samples. For each pair of PDX and patient samples, the correlation between sample-specific spatial cell type correlations is shown. The correlations are higher between PDX and LFS medulloblastomas as compared to PDX and sporadic medulloblastomas (two-sided Mann-Whitney U-test, n=96 (LFS), n=80 (sporadic)).

Supplementary Figure 20

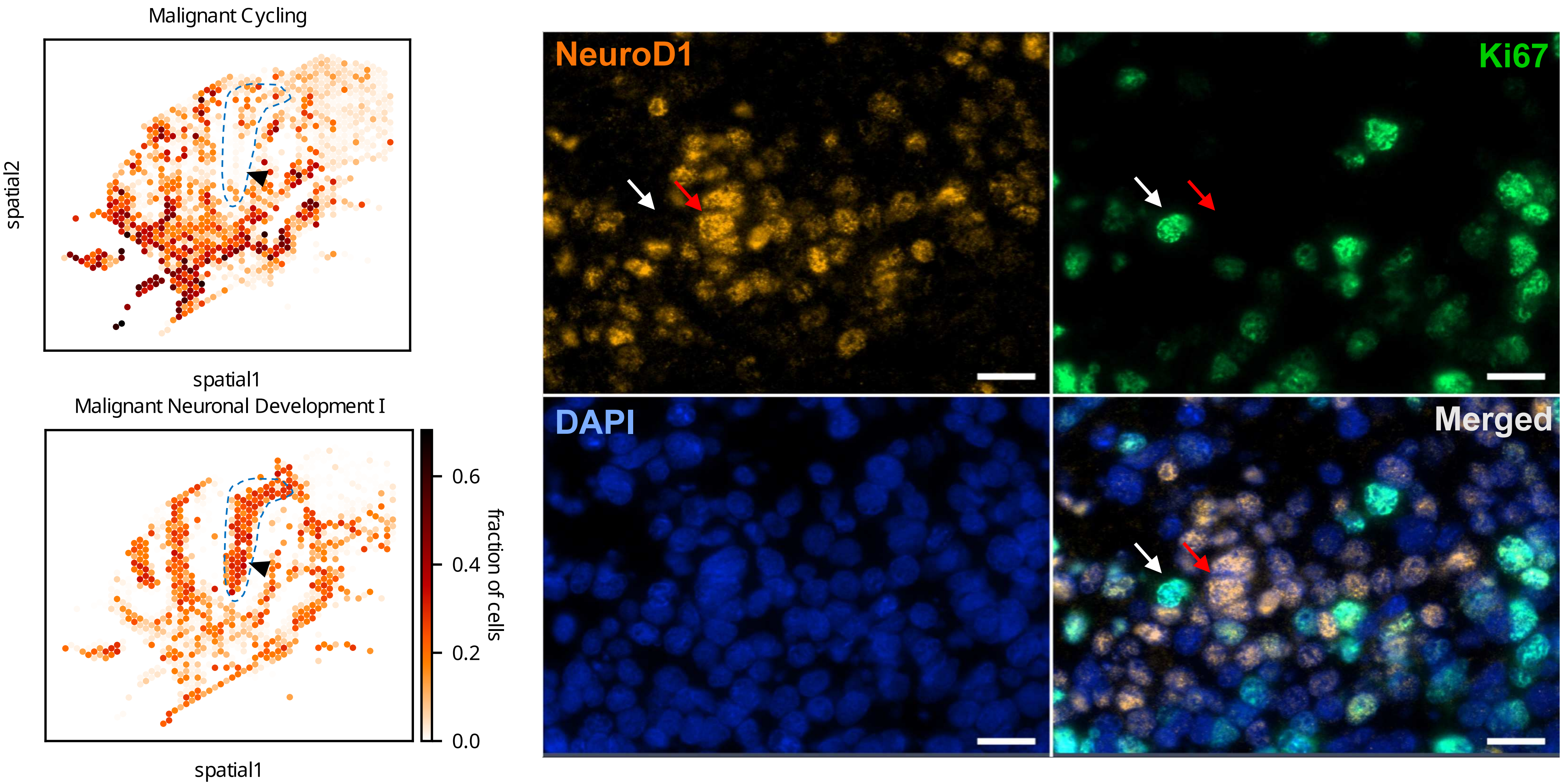

Supplementary Figure 20. Proliferating versus differentiated tumour cells in patient-derived xenografts: mutually exclusive transcriptional programs that recapitulate patient tumour signatures (1166 spots). Representative image of one PDX sample. Scale bar, 10 μm.

Supplementary Figure 21

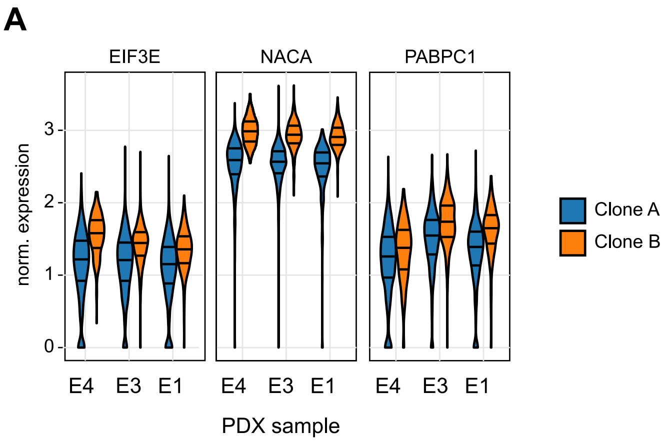

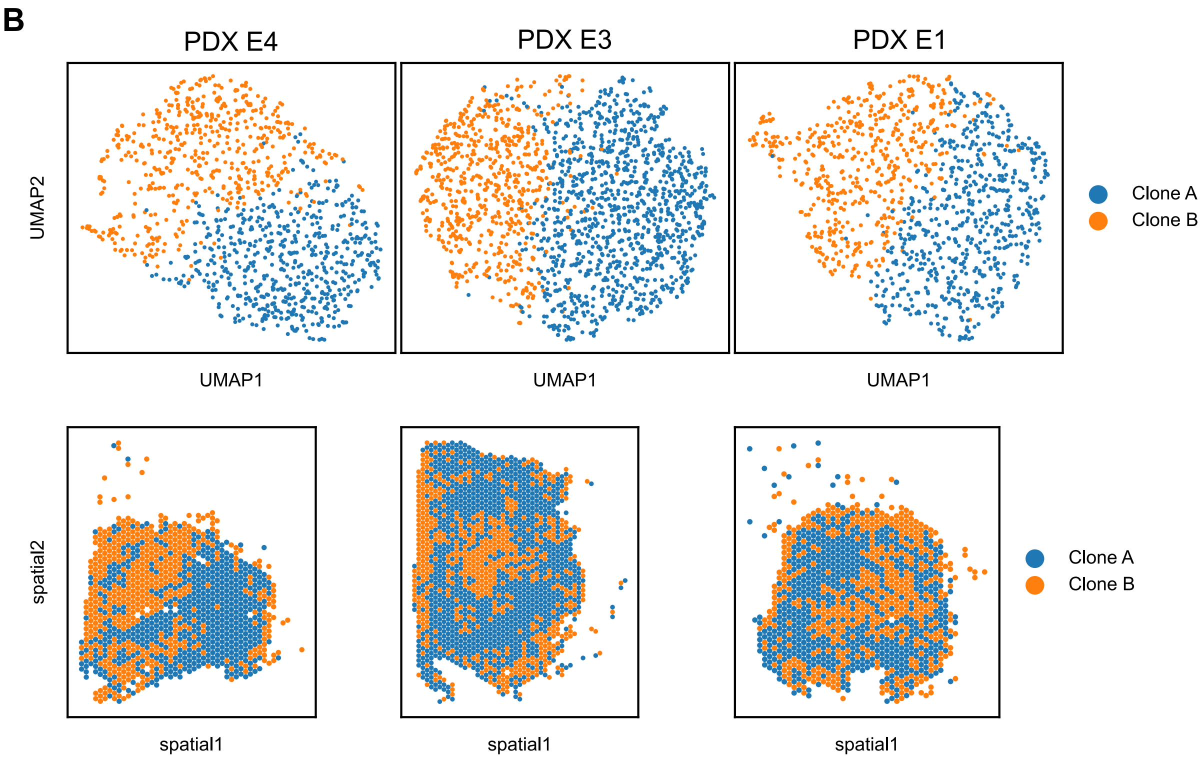

Supplementary Figure 21. Mapping genetic clones within the xenografted mouse brain tissue.

A. Mean expression of the highest-expressed genes with different copy numbers in the two clones in PDX samples PDX E1, PDX E3, PDX E4 Visium spots where a gene was not detected were excluded from the gene's mean calculation.

B. Leiden clustering of PDX samples PDX E1 (1147 spots), PDX E3 (1741 spots), PDX E4 (1105 spots) using only genes with different copy numbers in the two clones.

Supplementary Figure 22

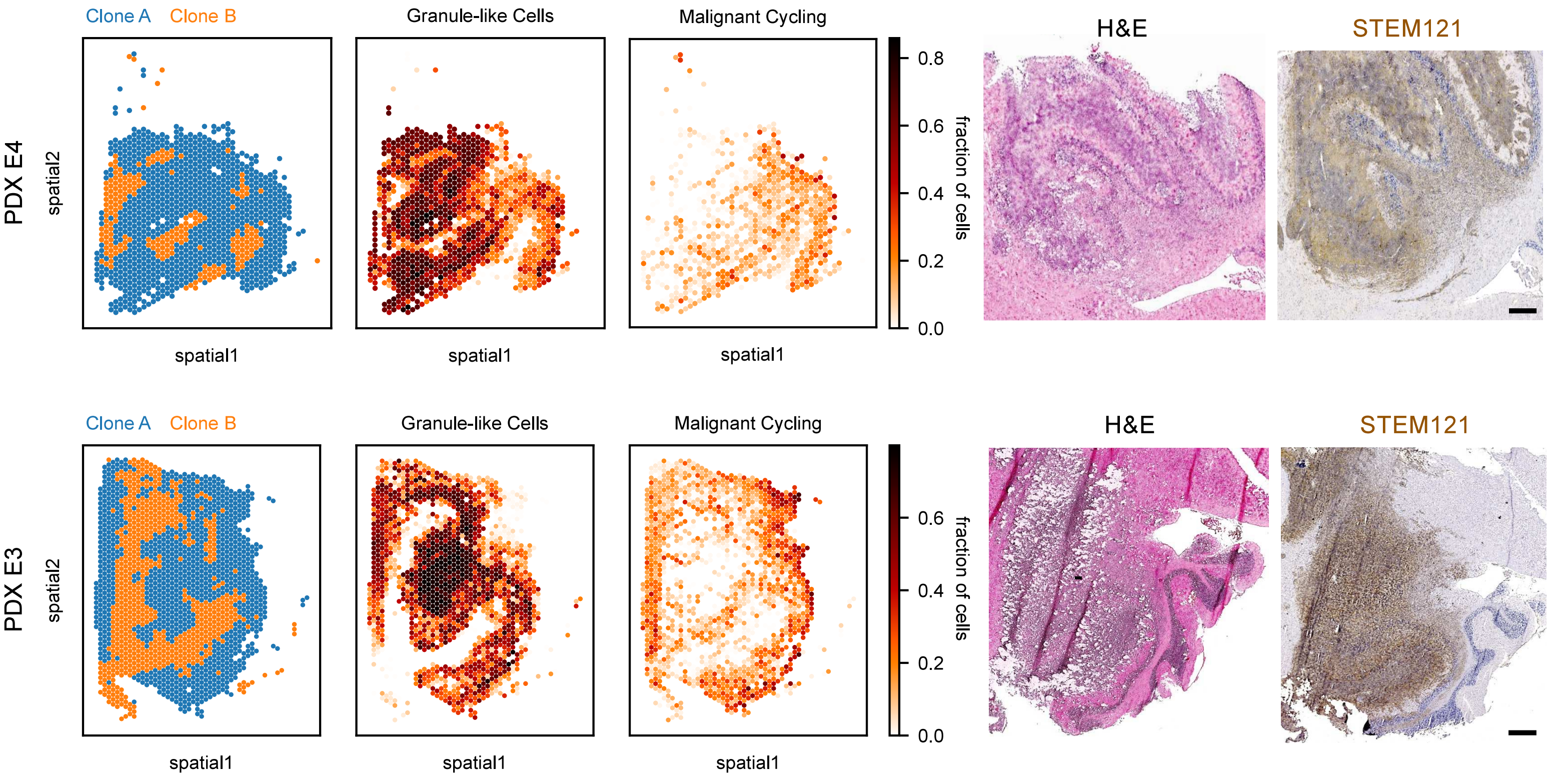

Supplementary Figure 22. Regrown tumours in PDX show the same major genetic clones as tumours before the treatment. 1105 spots (PDX E4), 1741 spots (PDX E3). STEM121 and hematoxylin and eosin staining in tissue sections of samples PDX E4 and PDX E3. Scale bar, 500 µm.

Supplementary Figure 23

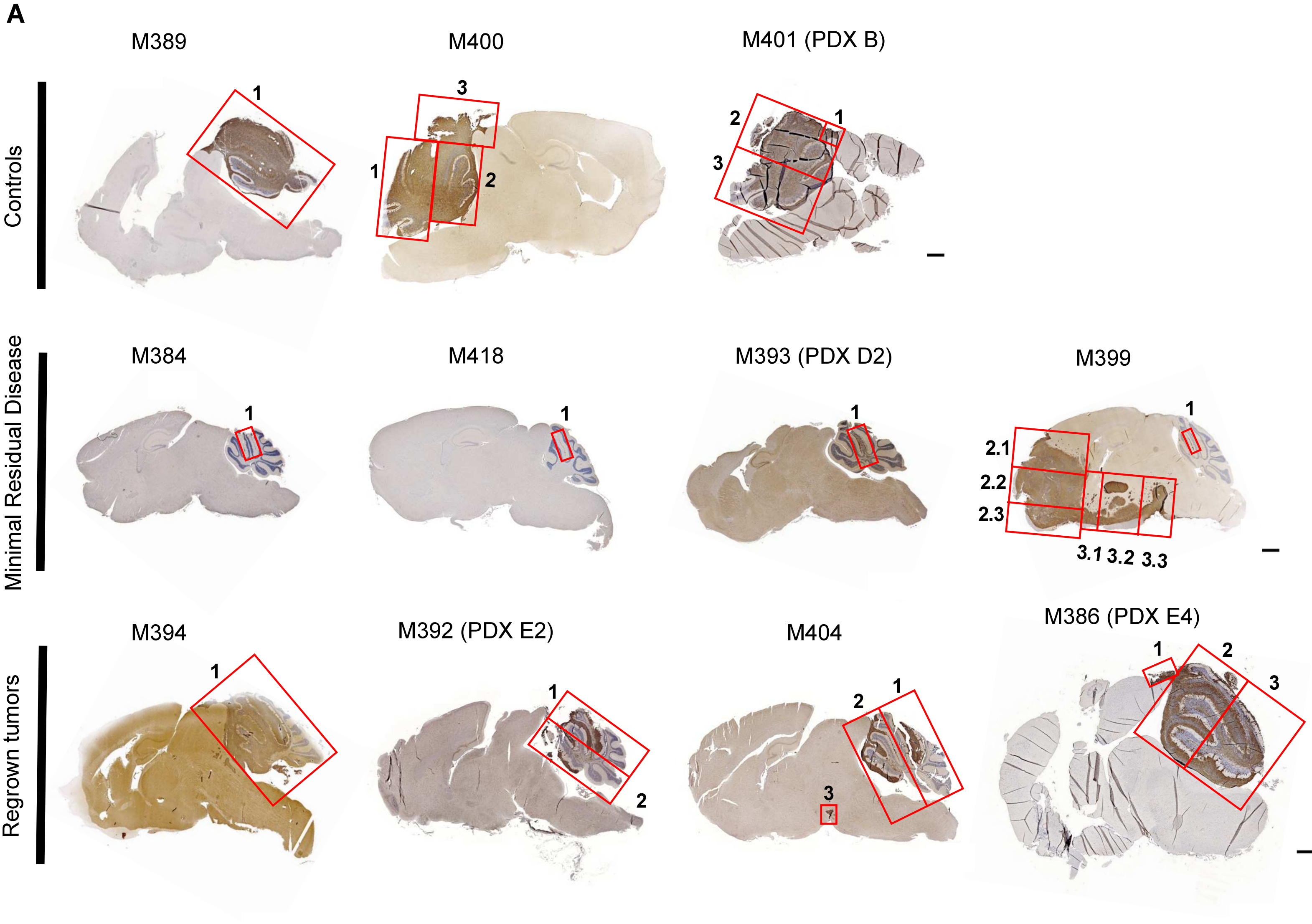

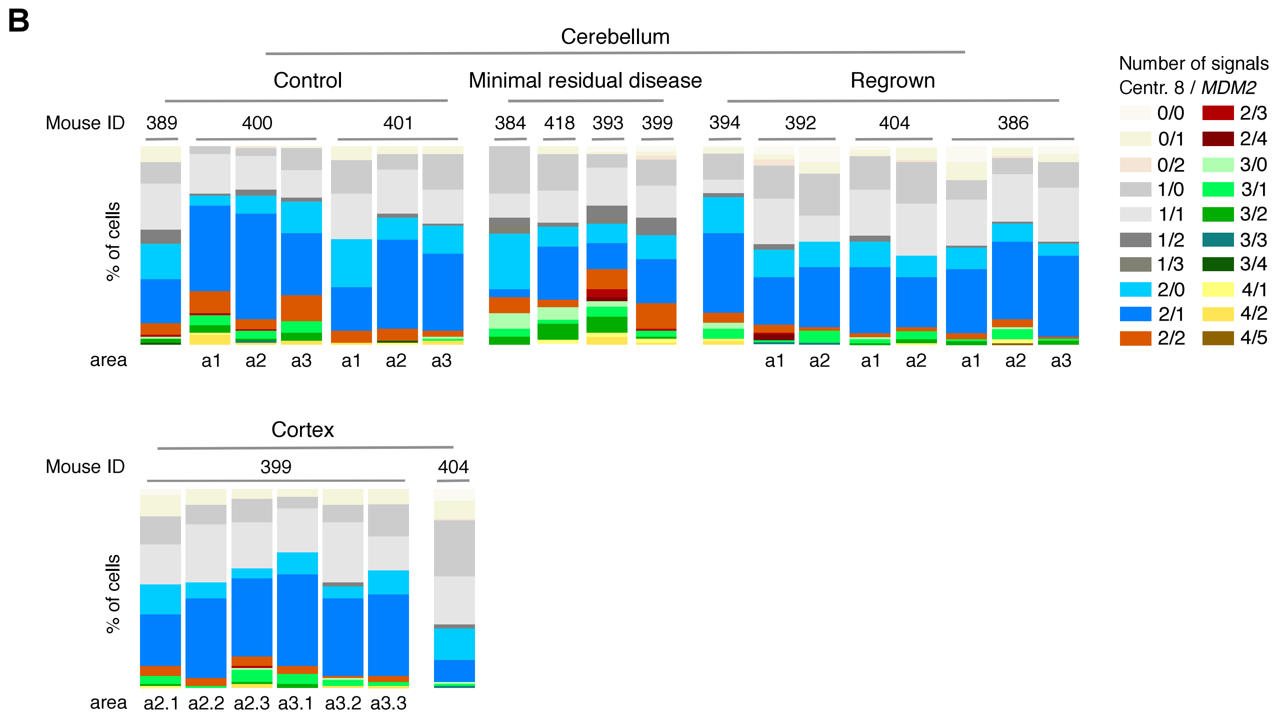

Supplementary Figure 23.

A. STEM121 stains for all 11 patient-derived-xenografts used for FISH and location of the areas used for the quantification of FISH signals. Scale bar, 500 µm.

B. Proportion of clones assessed by FISH (probes for *MDM2* and centromere 8) in control animals, at minimal residual disease and in regrown tumours in the cerebellum. Bottom, proportion of clones assessed by FISH in the non-irradiated cortex in mice for which invasion from the cerebellum to the cortex was observed. Numbers over the bars indicate animal IDs. a1, a2, and a3 indicate the brain regions in which the quantifications were made.

Supplementary Figure 24

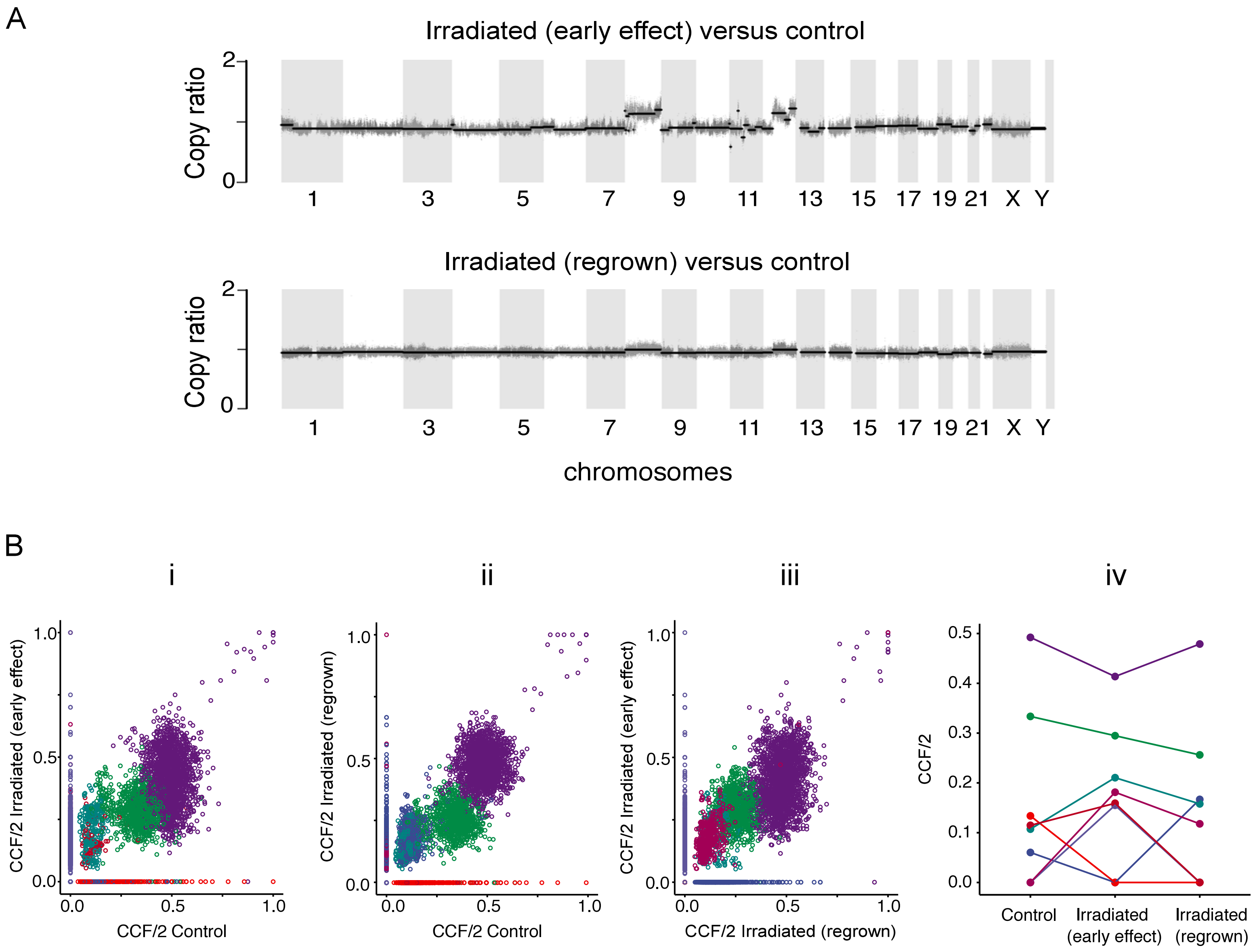

Supplementary Figure 24.

A. Whole-genome sequencing shows only minor changes in copy-number variants after treatment. Comparisons of copy-number variants are shown between carbon ion treated (early effect, 2 days after irradiation) and untreated control animals (top panel) and between carbon ion treated (regrown, 91 days after irradiation) and untreated control animals (lower panel), respectively.

B. Subclonal landscape of somatic SNVs in medulloblastoma PDX models: **(i)** Control (untreated) as compared to irradiated tumour 2 days after irradiation (early effect); **(ii)** Control as compared to irradiated tumour 91 days after irradiation (regrown); **(iii)** Irradiated tumour (early effect) as compared to regrown tumour long term after irradiation. Different colours highlight subclones as identified by binomial clustering with VIBER[3]. Measured variant allele frequencies (VAF) on all chromosomes were standardised between different copy numbers by conversion into pseudo-heterozygous VAFs (computed as CCF/2, where CCF is the cancer cell fraction). **(iv)** Evolution of subclonal size between tumours. Shown are the mean of the subclonal clusters as identified in i-iii for the medulloblastoma PDX Control (untreated), the PDX shortly after irradiation (early effect), and the PDX long term after irradiation (regrown).

Supplementary Figure 25

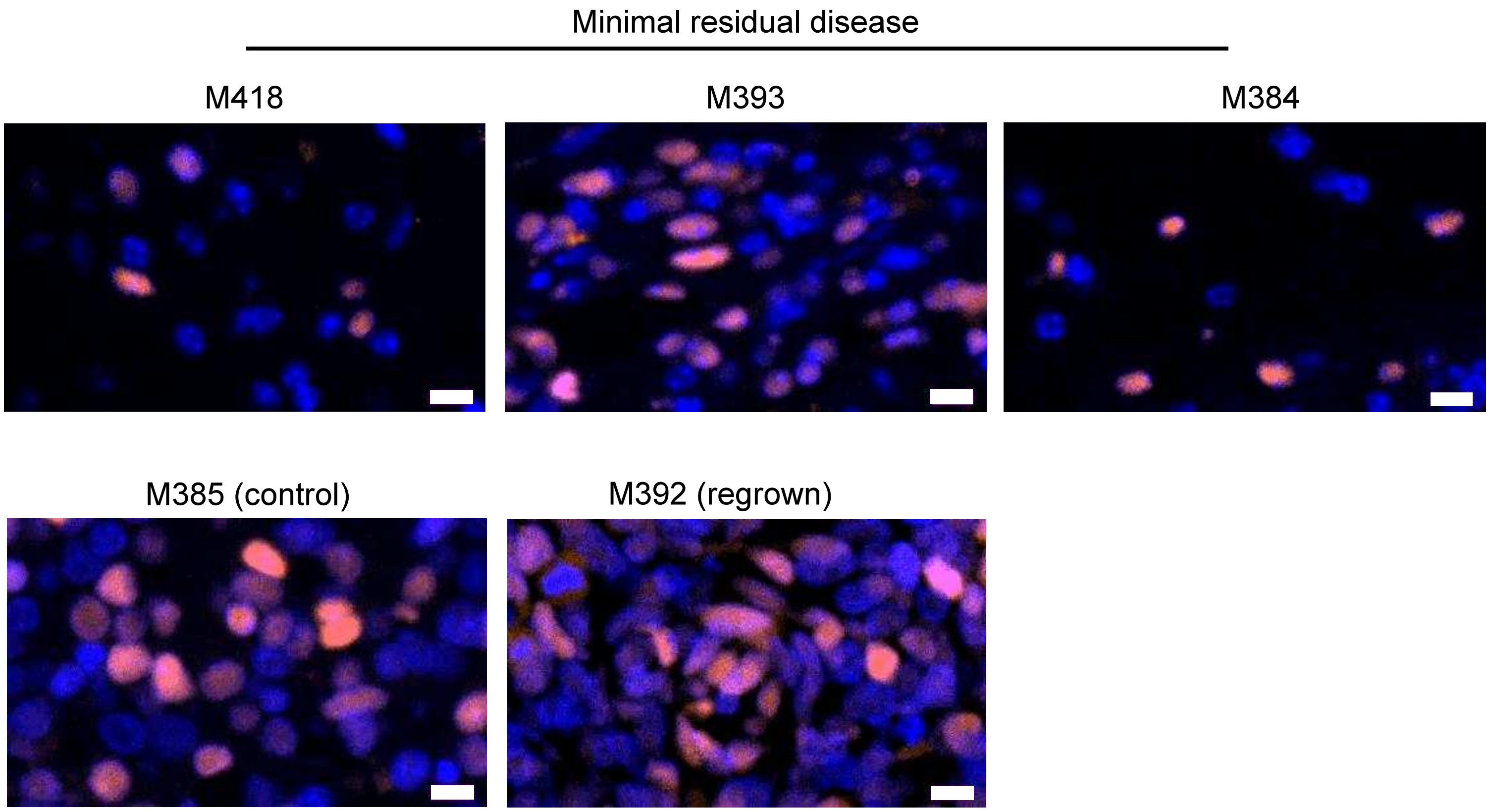

Supplementary Figure 25. SOX2 expression in PDX (immunofluorescence). The images are representative of five PDX samples with animal IDs stated on the image. Scale bar, 10 μm.

Supplementary Figure 26

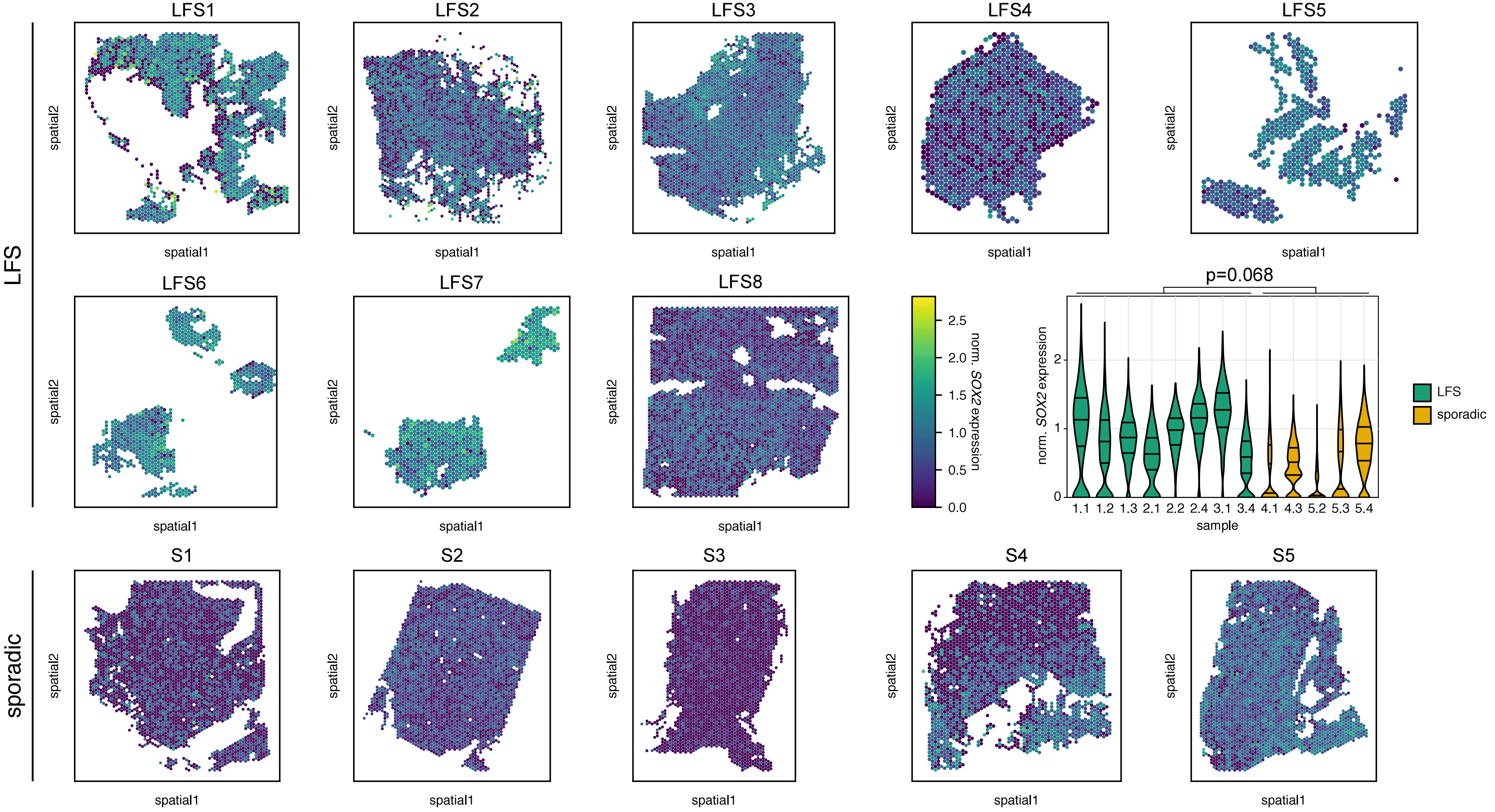

Supplementary Figure 26. *SOX2* expression in human medulloblastomas (Visium data). Statistical analysis was performed using DESeq2 with IHW multiple testing correction (n=8 (LFS), n=5 (sporadic)). 1590 spots (LFS1), 2642 spots (LFS2), 2469 spots (LFS3), 881 spots (LFS4), 500 spots (LFS5), 631 spots (LFS6), 537 spots (LFS7), 3983 spots (LFS8), 2966 spots (S1), 2621 spots (S2), 2251 spots (S3), 2198 spots (S4), 2723 spots (S5).

Supplementary Figure 27

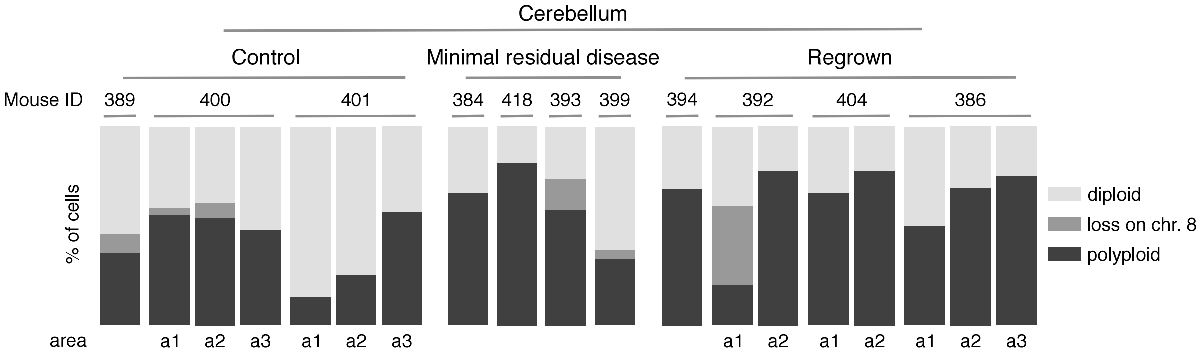

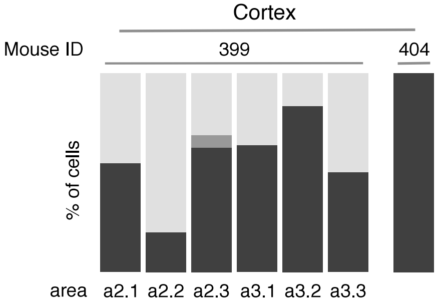

Supplementary Figure 27. Proportion of clones assessed by FISH (probes for MDM2 and centromere 8) in control animals, at minimal residual disease stage and in regrown tumours in the cerebellum. Bottom, proportion of clones assessed by FISH in the non-irradiated cortex in mice for which invasion from the cerebellum to the cortex was observed. Numbers over the bars indicate animal IDs. a1, a2, and a3 indicate the tumour regions in which the quantifications were made.

Supplementary Figure 28

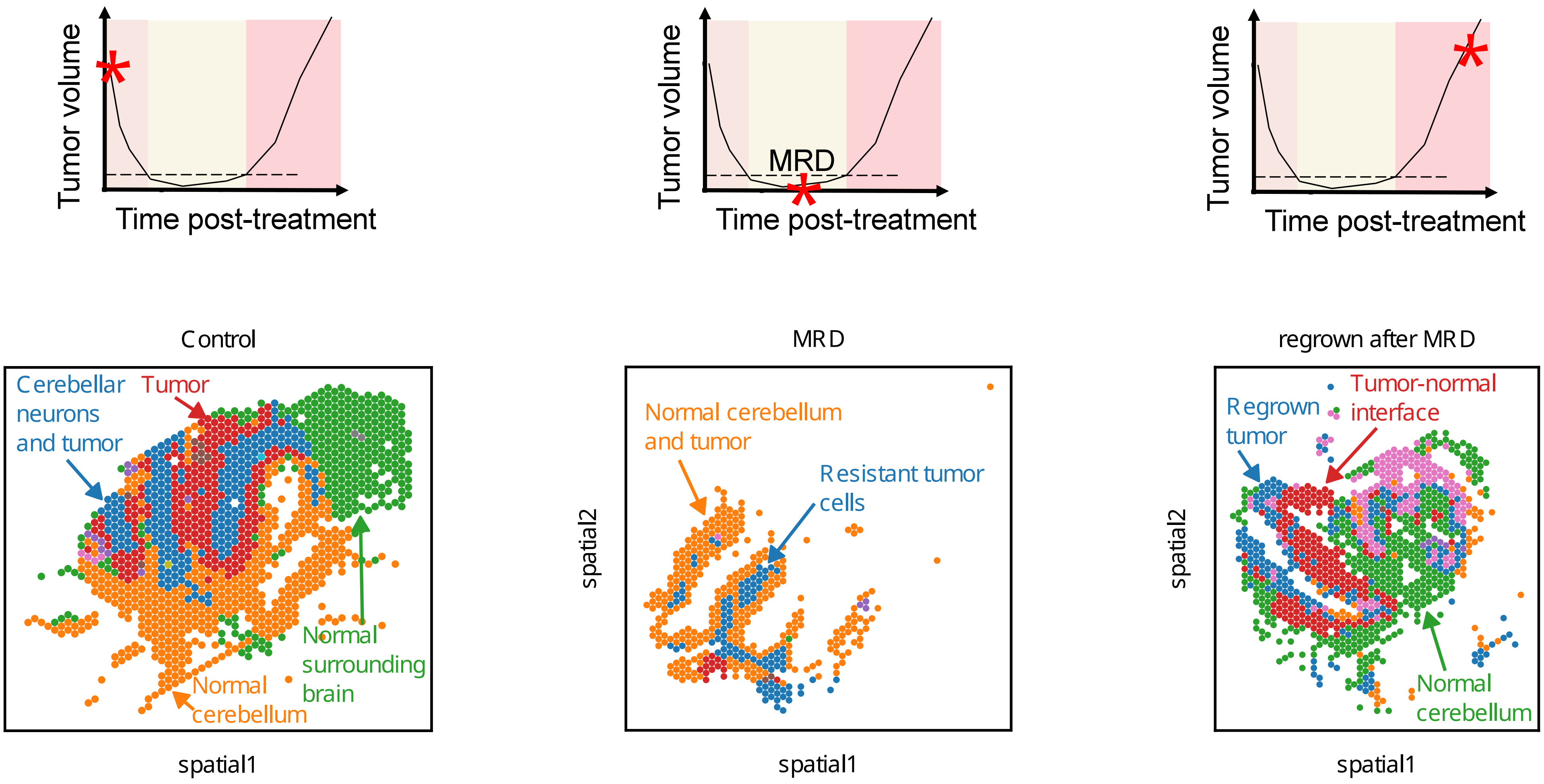

Supplementary Figure 28. Spatial gene expression analysis over the course of the PDX treatment. 3 samples are shown. 1166 spots (control), 416 spots (MRD), 936 spots (regrown).

Supplementary Figure 29

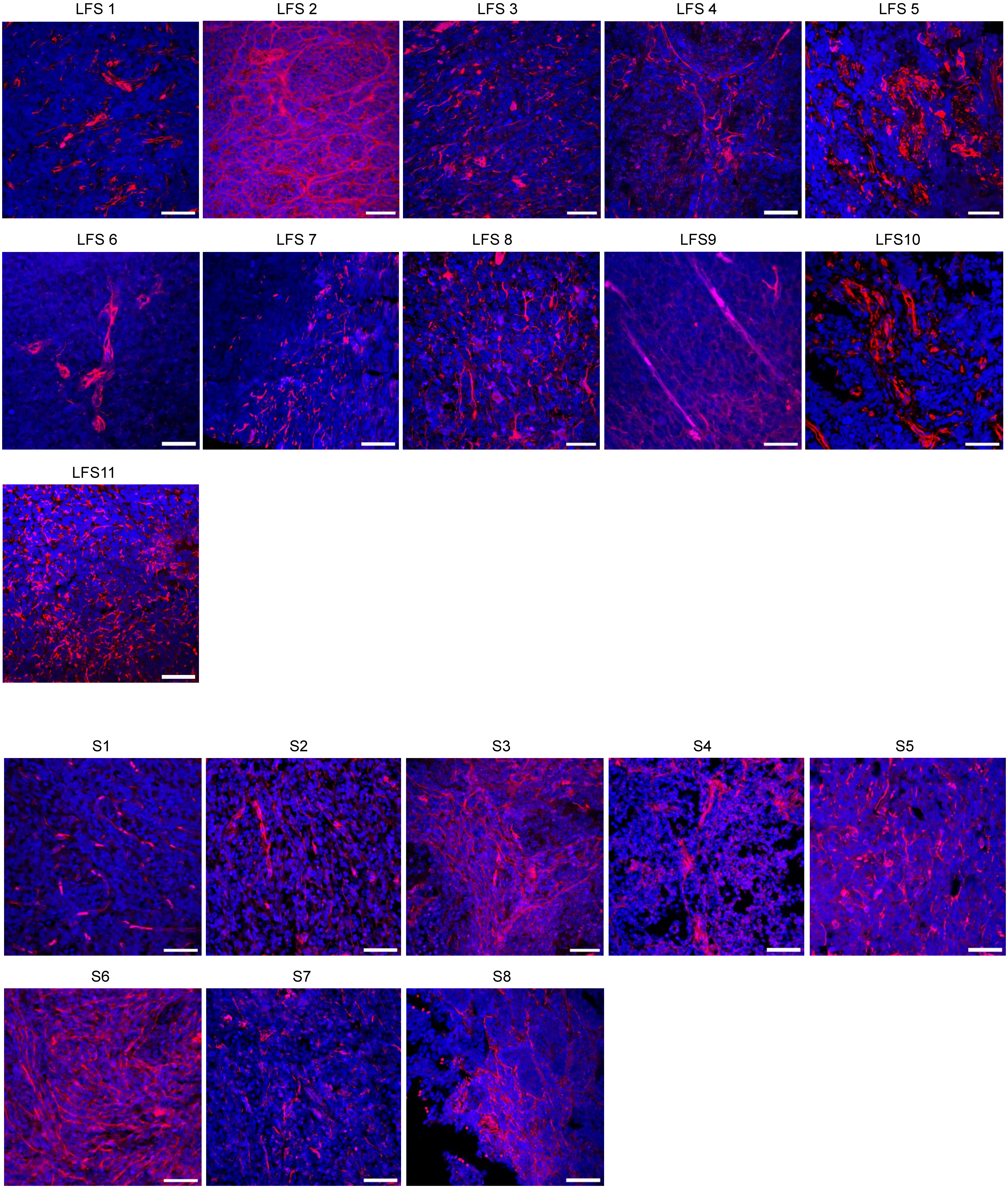

Supplementary Figure 29. Representative images of nestin expression (red signal, blue signal shows DAPI counterstain) visualised by immunofluorescence staining and confocal microscopy in tumour tissue sections from 11 LFS and 8 sporadic medulloblastoma patients. LFS1-LFS8 and S1-S5 are the patients for which spatial transcriptomics analysis was performed. Additional LFS and sporadic samples were used for immunofluorescence analysis. Scale bar, 50 µm.

Supplementary Figure 30

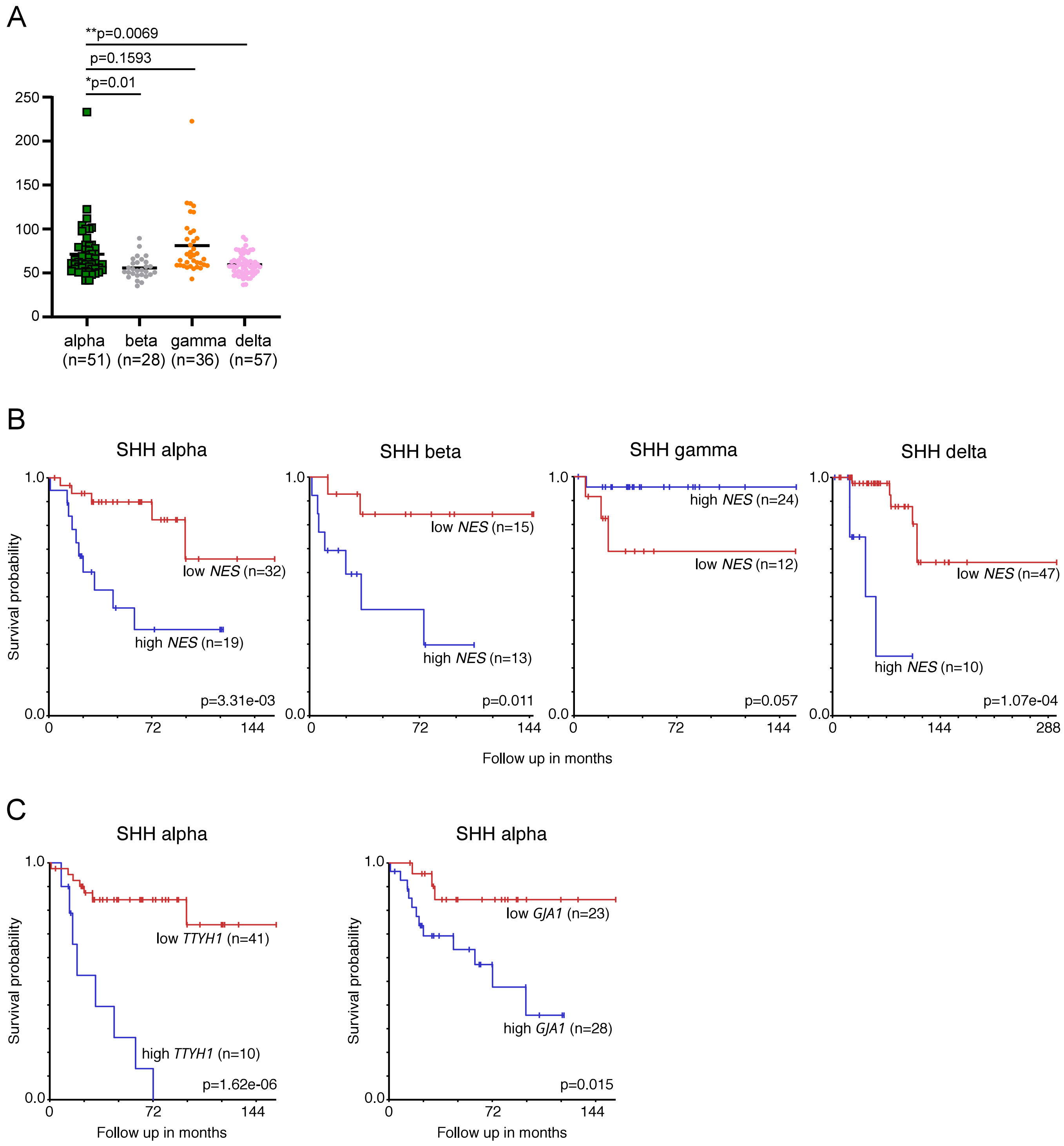

Supplementary Figure 30.

A. Nestin expression observed in bulk RNA sequencing data (R2 database, Tumour Medulloblastoma - Cavalli - 763 - rma_sketch - hugene11t dataset). Statistical analysis was done with two-tailed unpaired t-test.

B. Kaplan-Meier survival analysis in four SHH-MB subtypes grouped by *NES* expression (dataset as in A). High *NES* expression is associated with poor outcome in the SHH alpha, beta and delta subtypes (log-rank test). The SHH alpha subtype is enriched for LFS medulloblastomas.

C. Expression levels of tumour microtube network relevant markers such as *TTYH1* and *GJA1* are linked with clinical outcome in SHH medulloblastoma, with high expression levels significantly associated with shorter survival in the SHH alpha subgroup enriched for LFS medulloblastomas (expression data from the R2 database, log rank test). The total number of patients analyzed within each subtype is shown on the individual figure panels.

Supplementary Figure 31

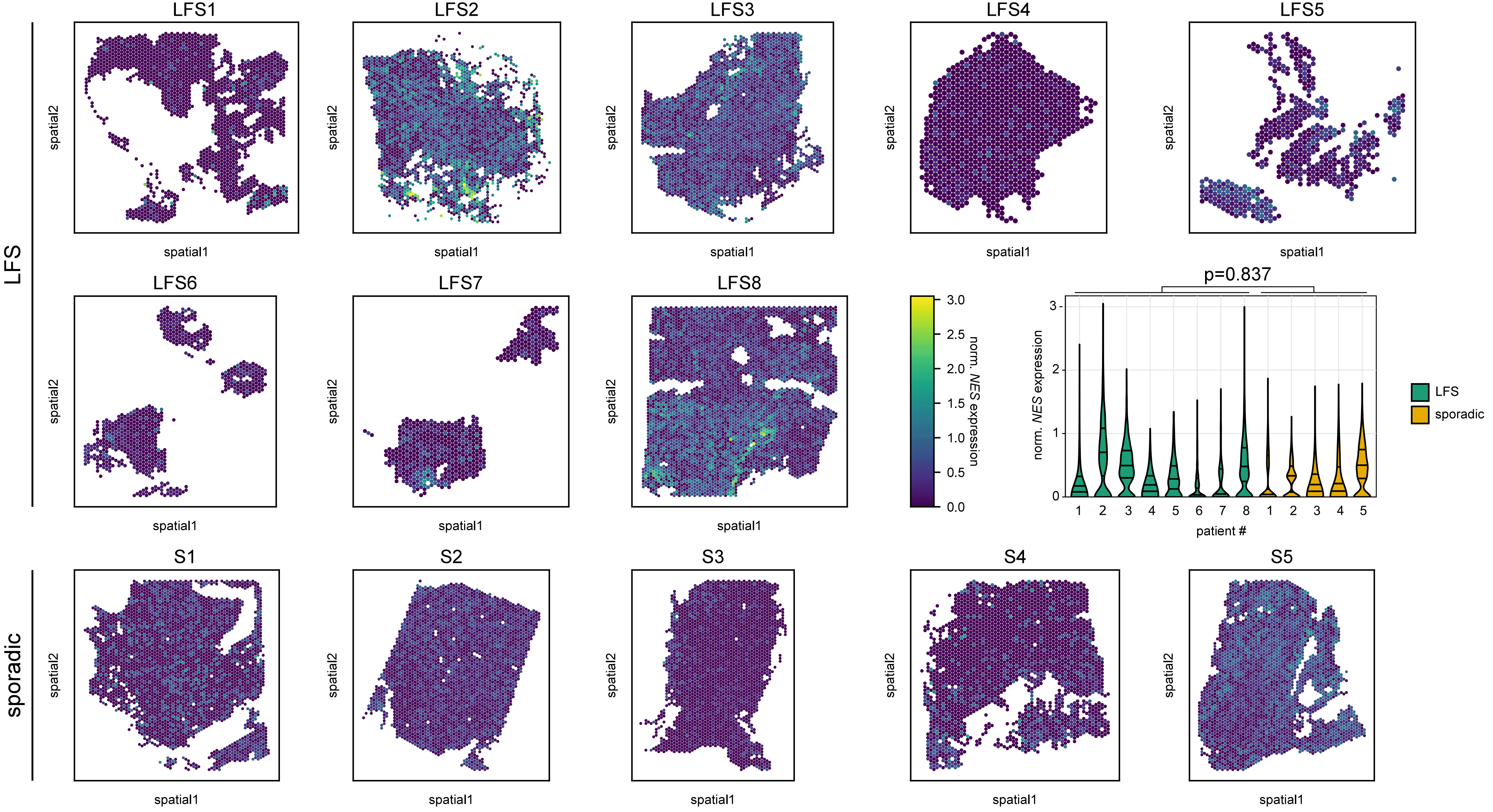

Supplementary Figure 31. Nestin expression in Visium human data. Statistical analysis was performed using DESeq2 with IHW multiple testing correction (n=8 (LFS), n=5 (sporadic)). 1590 spots (LFS1), 2642 spots (LFS2), 2469 spots (LFS3), 881 spots (LFS4), 500 spots (LFS5), 631 spots (LFS6), 537 spots (LFS7), 3983 spots (LFS8), 2966 spots (S1), 2621 spots (S2), 2251 spots (S3), 2198 spots (S4), 2723 spots (S5).

Supplementary Figure 32

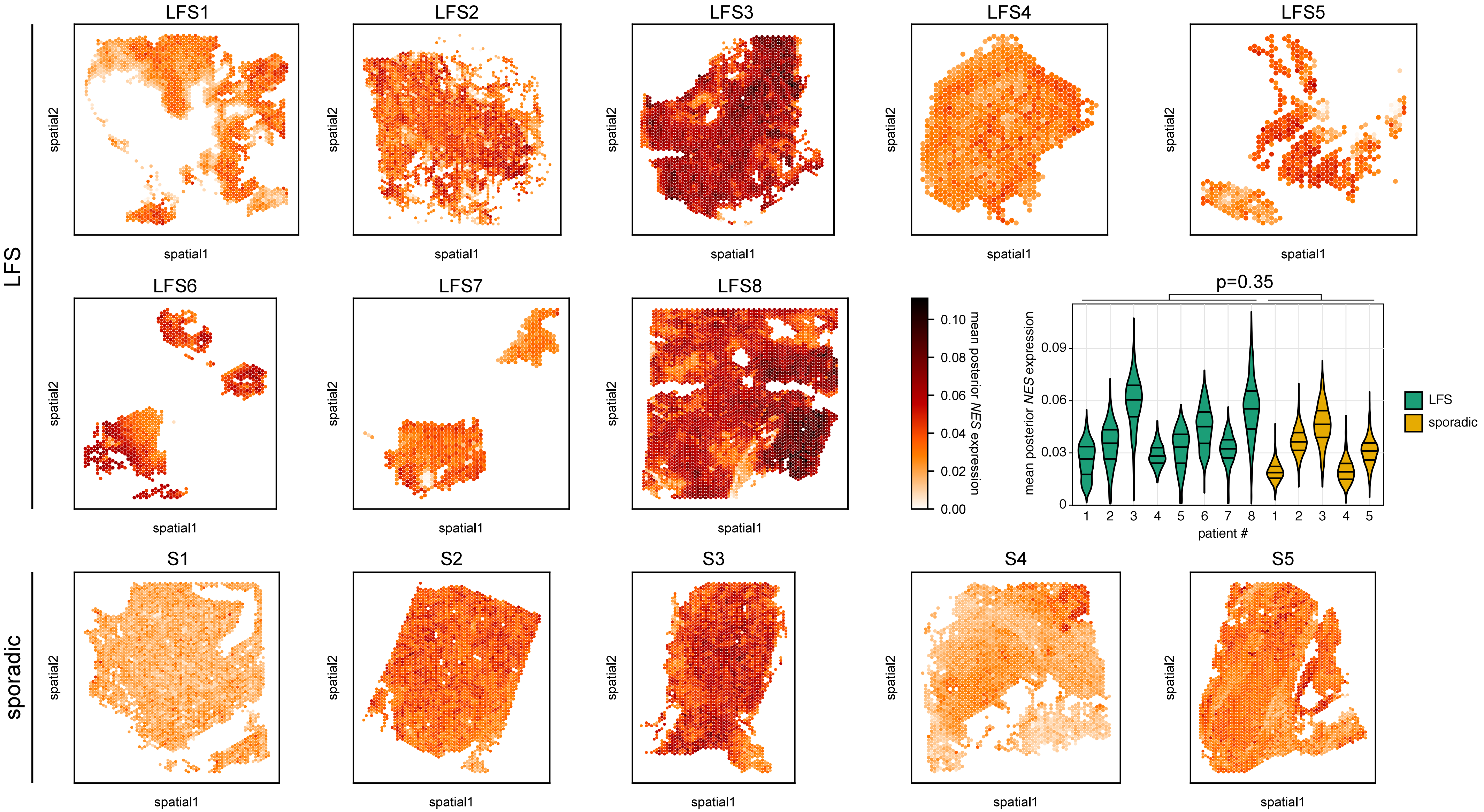

Supplementary Figure 32. Mean posterior Nestin expression in malignant cells (mean of the posterior distribution of Nestin expression after fitting the model) as inferred by Cell2location in Visium human data. Significance testing was performed using a two-sided Mann-Whitney U-test (n=8 (LFS), n=5 (sporadic)). 1590 spots (LFS1), 2642 spots (LFS2), 2469 spots (LFS3), 881 spots (LFS4), 500 spots (LFS5), 631 spots (LFS6), 537 spots (LFS7), 3983 spots (LFS8), 2966 spots (S1), 2621 spots (S2), 2251 spots (S3), 2198 spots (S4), 2723 spots (S5).

Supplementary Figure 33

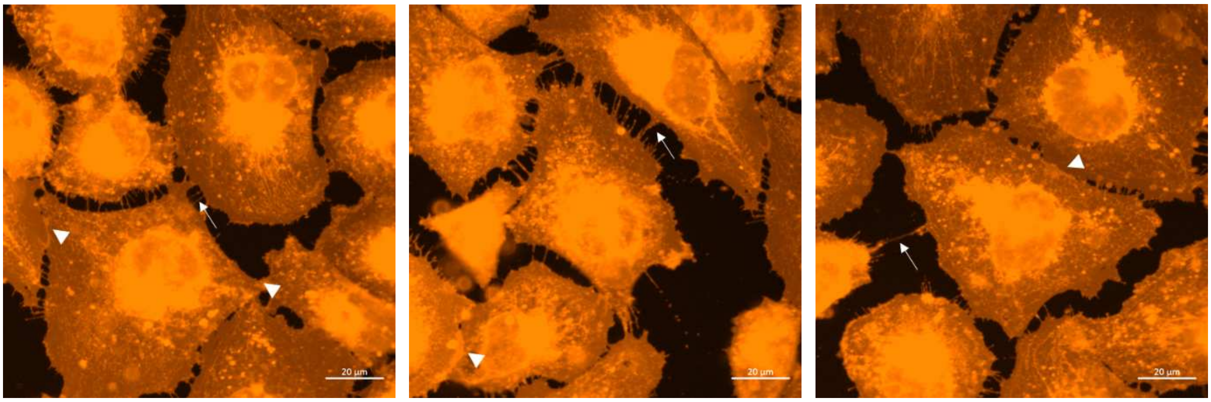

Supplementary Figure 33. Images of LipiLight560 and Rhod-2 staining illustrating membrane connections (arrowheads) and thin processes (arrows) in DAOY cells. The images are representative of three technical replicates.

Supplementary References

1. Cavalli, F. M. G. *et al*. Intertumoral Heterogeneity within Medulloblastoma Subgroups. *Cancer Cell* **31**, 737-754.e6 (2017).

2. Weinstein, J. N. *et al*. The Cancer Genome Atlas Pan-Cancer analysis project. *Nat Genet* **45**, 1113–20 (2013).

3. Caravagna, G. *et al*. Subclonal reconstruction of tumors by using machine learning and population genetics. *Nat Genet* **52**, 898–907 (2020).
