# Supplementary material for: Spatio-temporal transcriptomics of chromothriptic SHH-medulloblastoma identifies multiple genetic clones that resist treatment and drive relapse
Source: Nat Commun. 2024 Nov 29;15:10370. doi: 10.1038/s41467-024-54709-w (PMC11604656; doi:10.1038/s41467-024-54709-w)
Supplement: Supplementary file 2 — Description of Additional Supplementary Information [file 41467_2024_54709_MOESM2_ESM.docx]

**Description of Additional Supplementary Files**

File Name: Supplementary Data 1

Description: Patient characteristics

File Name: Supplementary Data 2

Description: P-values for the cell type abundance estimates shown in Fig.1D

File Name: Supplementary Data 3

Description: Fraction of T cells CD8 with fold change and p-values

File Name: Supplementary Data 4

Description: Fraction of T regs with fold change and pvalues

File Name: Supplementary Data 5

Description: Spatial variance analysis. Significance testing was performed using a twosided Mann-Whitney U-test with Benjamini-Hochberg multiple testing correction.

File Name: Supplementary Data 6

Description: Differential expression analysis between LFS and sporadic medulloblastomas. Analysis was performed with DESeq2, false discovery rate was controlled using the Benjamini-Hochberg procedure with independent hypothesis weighting.

File Name: Supplementary Data 7

Description: COMMOT ligand-receptor interactions

File Name: Supplementary Data 8

Description: P-values for the cell type fractions shown in Fig. 4F

File Name: Supplementary Data 9

Description: Overview of patient-derived xenografts

File Name: Supplementary Movie S1

Description: 5- minute video of DAOY cells grown in 2D monolayer assay demonstrating calcium activity with the Calbryte590 calcium indicator.

File Name: Supplementary Movie S2

Description: 5- minute video of DAOY cells grown in 2D monolayer assay demonstrating calcium activity with the Fluo-4 calcium indicator.

File Name: Supplementary Movie S3

Description: 5- minute video of DAOY cells grown in 2D monolayer assay demonstrating calcium activity with the Rhod2 calcium indicator.
