# Supplementary material for: Spatio-temporal transcriptomics of chromothriptic SHH-medulloblastoma identifies multiple genetic clones that resist treatment and drive relapse
Source: Nat Commun. 2024 Nov 29;15:10370. doi: 10.1038/s41467-024-54709-w (PMC11604656; doi:10.1038/s41467-024-54709-w)
Supplement: Supplementary file 15 — Reporting Summary [file 41467_2024_54709_MOESM15_ESM.pdf]

nature portfolio

Corresponding author(s): Oliver Stegle
Aurelie Ernst

Last updated by author(s): Nov 7, 2024

Reporting Summary

Nature Portfolio wishes to improve the reproducibility of the work that we publish. This form provides structure for consistency and transparency in reporting. For further information on Nature Portfolio policies, see our Editorial Policies and the Editorial Policy Checklist.

Statistics

For all statistical analyses, confirm that the following items are present in the figure legend, table legend, main text, or Methods section.

n/a	Confirmed	
☐	☒	The exact sample size (*n*) for each experimental group/condition, given as a discrete number and unit of measurement
☐	☒	A statement on whether measurements were taken from distinct samples or whether the same sample was measured repeatedly
☐	☒	The statistical test(s) used AND whether they are one- or two-sided<br>*Only common tests should be described solely by name; describe more complex techniques in the Methods section.*
☐	☒	A description of all covariates tested
☐	☒	A description of any assumptions or corrections, such as tests of normality and adjustment for multiple comparisons
☐	☒	A full description of the statistical parameters including central tendency (e.g. means) or other basic estimates (e.g. regression coefficient) AND variation (e.g. standard deviation) or associated estimates of uncertainty (e.g. confidence intervals)
☐	☒	For null hypothesis testing, the test statistic (e.g. *F*, *t*, *r*) with confidence intervals, effect sizes, degrees of freedom and *P* value noted<br>*Give P values as exact values whenever suitable.*
☒	☐	For Bayesian analysis, information on the choice of priors and Markov chain Monte Carlo settings
☒	☐	For hierarchical and complex designs, identification of the appropriate level for tests and full reporting of outcomes
☒	☐	Estimates of effect sizes (e.g. Cohen's *d*, Pearson's *r*), indicating how they were calculated

Our web collection on statistics for biologists contains articles on many of the points above.

Software and code

Policy information about availability of computer code

Data collection — *Provide a description of all commercial, open source and custom code used to collect the data in this study, specifying the version used OR state that no software was used.*

Data analysis

For mapping and counting of sequeincing reads from 10x Visium experiments, we used SpaceRanger 1.3.1 (10x Genomics). Sorting of sequencing reads from PDX mouse models was done with xengsort 1.1.0 (https://gitlab.com/genomeinformatics/xengsort). Further analysis was performed in Python using scanpy 1.9.1 (https://github.com/scverse/scanpy) . Cell type deconvolution was performed using Cell2location 0.1 (https://github.com/BayraktarLab/cell2location). Cell-cell communication was analyzed with COMMOT 0.0.2 (https://github.com/zcang/COMMOT). Spatial clustering and estimation of spatial variance was performed with SpatialDE2 (https://github.com/PMBio/spatialde). Integration of PDX samples was done with scVI 0.16.2 (https://github.com/scverse/scvi-tools). Differential gene expression analysis was performed with DESeq2 1.36.0 (http://bioconductor.org/packages/release/bioc/html/DESeq2.html) and IHW 1.30.0 (https://bioconductor.org/packages/release/bioc/html/IHW.html). Nucleus segmentation was performed with Cellpose 2.2.2 (https://www.cellpose.org/). Analysis code written for this manuscript is available at https://github.com/ilia-kats/medulloblastoma-paper.

For immune cell deconvolution analysis, we used cibersortX (https://cibersortx.stanford.edu/) in October 2022. For copy number analysis, GATK 4.1.3.0 was used.

The Heidelberg Brain Tumor Methylation Classifier v11b6 (https://www.molecularneuropathology.org) was applied for molecular classification. Copy-number variation analysis from 450k and EPIC methylation array data was performed using the conumee Bioconductor package version 1.12.0.

For quantification of Immunofluorescence Imagej (version 2.0) was used. For tumor microtubes, software AIVIA (version 10.1) was used in addition. 2D calcium imaging was done using a Zeiss LSM 980 Airyscan NIR confocal microscope and each time series was recorded over a 35-minute period. FISH quantification was done manually under Axio Zeiss Imager.M2 microscope.

MRI tumour volume was determined by manual segmentation using Bruker ParaVision software 6.0.1.

For manuscripts utilizing custom algorithms or software that are central to the research but not yet described in published literature, software must be made available to editors and reviewers. We strongly encourage code deposition in a community repository (e.g. GitHub). See the Nature Portfolio guidelines for submitting code & software for further information.

Data

Policy information about availability of data

All manuscripts must include a data availability statement. This statement should provide the following information, where applicable:

- Accession codes, unique identifiers, or web links for publicly available datasets
- A description of any restrictions on data availability
- For clinical datasets or third party data, please ensure that the statement adheres to our policy

The sequencing data generated in this study have been deposited at the European Genome-phenome Archive with accession number EGAS00001007128 (https://ega-archive.org/studies/EGAS00001007128). The data is available under restricted access because partly personal data cannot be publicly available, due to the European General Data Protection Regulation (GDPR) and the German General Data Protection Regulation (GDPR) and to respect the patient consent forms. For data sharing, a Data Transfer Agreement (DTA) has to be legally settled between the requesting institute and the providing institute. Once the data access has been granted, the access is usually available for 5 years, depending on the individual patient consent forms. We will do our best to process the requests and get DTAs in place as fast as possible.

The remaining data are available within the Supplementary Information or Source Data file.

Research involving human participants, their data, or biological material

Policy information about studies with human participants or human data. See also policy information about sex, gender (identity/presentation), and sexual orientation and race, ethnicity and racism.

Reporting on sex and gender	We report the sex of the participants in Supplementary Table S1
Reporting on race, ethnicity, or other socially relevant groupings	We do not have information on race/ethniticity/etc.
Population characteristics	Our study is not on population-scale
Recruitment	Clinical data and tissue of all cases in the study were collected after receiving written informed consent from the respective patients or their legal representatives and after approval by the ethic board of Heidelberg University. Participants did not receive compensation and were informed of this prior to enrolment in the study.
Ethics oversight	Ethic boards of Heidelberg University

Note that full information on the approval of the study protocol must also be provided in the manuscript.

Field-specific reporting

Please select the one below that is the best fit for your research. If you are not sure, read the appropriate sections before making your selection.

[x] Life sciences [ ] Behavioural & social sciences [ ] Ecological, evolutionary & environmental sciences

For a reference copy of the document with all sections, see nature.com/documents/nr-reporting-summary-flat.pdf

Life sciences study design

All studies must disclose on these points even when the disclosure is negative.

Sample size	No statistical method was used to predetermine sample size for spatial transcriptomics experiment.
Data exclusions	One PDX sample was excluded from the analyses due to very low tumor volume covering <100 Visium spots. Two human samples were excluded due to very poor data quality.
Replication	For human data, we have with two exceptions (LFS2/LFS3/LFS8 and LFS6/LFS7) one sample per patient. For the patient with multiple samples, the samples are from different time points and/or different tumour areas. For PDX samples, we generally have multiple samples from different mice for each time point and treatment regime.
Randomization	All animals used for treatment experiments with carbon ions and PARPi were randomised into treatment groups based on tumor volume measurements.
Blinding	The investigators were not blinded during animal follow up and outcome assessment.

Behavioural & social sciences study design

All studies must disclose on these points even when the disclosure is negative.

Study description	*Briefly describe the study type including whether data are quantitative, qualitative, or mixed-methods (e.g. qualitative cross-sectional, quantitative experimental, mixed-methods case study).*
Research sample	*State the research sample (e.g. Harvard university undergraduates, villagers in rural India) and provide relevant demographic information (e.g. age, sex) and indicate whether the sample is representative. Provide a rationale for the study sample chosen. For studies involving existing datasets, please describe the dataset and source.*
Sampling strategy	*Describe the sampling procedure (e.g. random, snowball, stratified, convenience). Describe the statistical methods that were used to predetermine sample size OR if no sample-size calculation was performed, describe how sample sizes were chosen and provide a rationale for why these sample sizes are sufficient. For qualitative data, please indicate whether data saturation was considered, and what criteria were used to decide that no further sampling was needed.*
Data collection	*Provide details about the data collection procedure, including the instruments or devices used to record the data (e.g. pen and paper, computer, eye tracker, video or audio equipment) whether anyone was present besides the participant(s) and the researcher, and whether the researcher was blind to experimental condition and/or the study hypothesis during data collection.*
Timing	*Indicate the start and stop dates of data collection. If there is a gap between collection periods, state the dates for each sample cohort.*
Data exclusions	*If no data were excluded from the analyses, state so OR if data were excluded, provide the exact number of exclusions and the rationale behind them, indicating whether exclusion criteria were pre-established.*
Non-participation	*State how many participants dropped out/declined participation and the reason(s) given OR provide response rate OR state that no participants dropped out/declined participation.*
Randomization	*If participants were not allocated into experimental groups, state so OR describe how participants were allocated to groups, and if allocation was not random, describe how covariates were controlled.*

Ecological, evolutionary & environmental sciences study design

All studies must disclose on these points even when the disclosure is negative.

Study description	*Briefly describe the study. For quantitative data include treatment factors and interactions, design structure (e.g. factorial, nested, hierarchical), nature and number of experimental units and replicates.*
Research sample	*Describe the research sample (e.g. a group of tagged Passer domesticus, all Stenocereus thurberi within Organ Pipe Cactus National Monument), and provide a rationale for the sample choice. When relevant, describe the organism taxa, source, sex, age range and any manipulations. State what population the sample is meant to represent when applicable. For studies involving existing datasets, describe the data and its source.*
Sampling strategy	*Note the sampling procedure. Describe the statistical methods that were used to predetermine sample size OR if no sample-size calculation was performed, describe how sample sizes were chosen and provide a rationale for why these sample sizes are sufficient.*
Data collection	*Describe the data collection procedure, including who recorded the data and how.*

Timing and spatial scale	*Indicate the start and stop dates of data collection, noting the frequency and periodicity of sampling and providing a rationale for these choices. If there is a gap between collection periods, state the dates for each sample cohort. Specify the spatial scale from which the data are taken*
Data exclusions	*If no data were excluded from the analyses, state so OR if data were excluded, describe the exclusions and the rationale behind them, indicating whether exclusion criteria were pre-established.*
Reproducibility	*Describe the measures taken to verify the reproducibility of experimental findings. For each experiment, note whether any attempts to repeat the experiment failed OR state that all attempts to repeat the experiment were successful.*
Randomization	*Describe how samples/organisms/participants were allocated into groups. If allocation was not random, describe how covariates were controlled. If this is not relevant to your study, explain why.*
Blinding	*Describe the extent of blinding used during data acquisition and analysis. If blinding was not possible, describe why OR explain why blinding was not relevant to your study.*

Did the study involve field work? ☐ Yes ☐ No

Field work, collection and transport

Field conditions	*Describe the study conditions for field work, providing relevant parameters (e.g. temperature, rainfall).*
Location	*State the location of the sampling or experiment, providing relevant parameters (e.g. latitude and longitude, elevation, water depth).*
Access & import/export	*Describe the efforts you have made to access habitats and to collect and import/export your samples in a responsible manner and in compliance with local, national and international laws, noting any permits that were obtained (give the name of the issuing authority, the date of issue, and any identifying information).*
Disturbance	*Describe any disturbance caused by the study and how it was minimized.*

Reporting for specific materials, systems and methods

We require information from authors about some types of materials, experimental systems and methods used in many studies. Here, indicate whether each material, system or method listed is relevant to your study. If you are not sure if a list item applies to your research, read the appropriate section before selecting a response.

Materials & experimental systems

n/a	Involved in the study	
☐	☒	Antibodies
☐	☒	Eukaryotic cell lines
☒	☐	Palaeontology and archaeology
☐	☒	Animals and other organisms
☒	☐	Clinical data
☒	☐	Dual use research of concern
☒	☐	Plants

Methods

n/a	Involved in the study	
☒	☐	ChIP-seq
☒	☐	Flow cytometry
☐	☒	MRI-based neuroimaging

Antibodies

Antibodies used

Ki67 #11882, Cell Signaling (dilution 1:50)

Ki67 #M7240 DAKO (dilution 1:100)

NeuroD1 #AF2746, Biotechne (dilution 1:50)

SOX2 #23064, Cell Signalling (dilution 1:200)

Nestin #ab105389, Abcam (dilution 1:100)

TOM20 #11802-1-AP, Proteintech (dilution 1:400)

Nestin, #NB100-1604, Novus Biologicals (dilution 1:500)

	STEM121 #Y40410, Takara (dilution 1:5000)<br><br>Secondary ABs:<br><br>Cy-3 Donkey anti-goat IgG #705-165-003, Jackson ImmunoCy3 (dilution 1:500)<br><br>Cy-5 Donkey anti-mouse IgG #715-175-151, Jackson Immuno (dilution 1:500)<br><br>AF 594 Donkey anti-rabbit IgG #A21207, Invitrogen (dilution 1:500)<br><br>CF™ 488A Donkey Anti-Chicken IgY #SAB4600031, Sigma Aldrich (dilution 1:500)
Validation	Ki67 #11882, Cell Signaling; tested by manufacturer for direct flow cytometric and immunofluorescent analysis in human cells.<br><br>Ki67 #M7240, DAKO Agilent; Manufacturer's website states: "In Western blotting of lysates of the multiple myeloma cell line, IM-9, the MIB-1 antibody labels bands of 345 and 395 kDa, identical to the bands labeled by the original Ki-67 antibody. Furthermore, Western blotting and competitive binding experiments clearly demonstrate that MIB-1, like the original Ki-67 antibody, reacts with an epitope encoded by a 66 bp repetitive element in the Ki-67 gene. In IHC the MIB-1 and the Ki-67 antibodies provide identical staining patterns on serial tonsillar frozen sections. The MIB-1 antibody recognizes native Ki-67 antigen and recombinant fragments of the Ki-67 molecule."<br><br>NeuroD1 #AF2746, Biotechne; validated according to manufacturer's website in ββTC-6 Mouse Cell Line<br><br>SOX2 #23064, Cell Signalling Technology; according to manufacturer, this antibody has been validated using SimpleChIP® Enzymatic Chromatin IP Kits.<br><br>Nestin #ab105389 Abcam; Knockout validated by manufacturer.<br><br>TOM20 #11802-1-AP, Proteintech; KD/KO validated by manufacturer.<br><br>Nestin, #NB100-1604, Novus Biologicals; Manufacturer lists publications tested in 4 confirmed species (Human, Mouse, Rat, Monkey) and publications tested in 7 applications (Flow, ICC, ICC/IF, IHC, IHC-Fr, IHC-P, WB)<br><br>STEM121 #Y40410, Takara; Manufacturer lists citations using this antibody for IHC and IF.<br><br>Secondary ABs:<br><br>Cy-3 Donkey anti-goat IgG #705-165-003, Jackson Immuno; Manufacturer's website states: "Based on immunoelectrophoresis and/or ELISA, the antibody reacts with whole molecule goat IgG. It also reacts with the light chains of other goat immunoglobulins. No antibody was detected against non-immunoglobulin serum proteins. The antibody may cross-react with immunoglobulins from other species."<br><br>Cy-5 Donkey anti-mouse IgG #715-175-151, Jackson Immuno; Manufacturer's website states: "Based on immunoelectrophoresis and/or ELISA, the antibody reacts with whole molecule mouse IgG. It also reacts with the light chains of other mouse immunoglobulins. No antibody was detected against non-immunoglobulin serum proteins. The antibody has been tested by ELISA and/or solid-phase adsorbed to ensure minimal cross-reaction with bovine, chicken, goat, guinea pig, syrian hamster, horse, human, rabbit, rat and sheep serum proteins, but it may cross-react with immunoglobulins from other species."<br><br>AF 594 Donkey anti-rabbit IgG #A21207, Invitrogen; validated in IF/ICC according to manufacturer's website.<br><br>CF™ 488A Donkey Anti-Chicken IgY #SAB4600031, Sigma Aldrich; used in immunohistochemistry acccording to manufacturer's website.

Eukaryotic cell lines

Policy information about cell lines and Sex and Gender in Research

Cell line source(s)	DAOY cells (ATCC® HTB-186™) were obtained from the American Type Culture Collection.
Authentication	The cells were authenticated by Single Nucleotide Polymorphism (SNP)-profiling (Multiplexion).
Mycoplasma contamination	The cells were confirmed to be negative for Mycoplasma contamination.
Commonly misidentified lines<br>(See ICLAC register)	No commonly misidentified cell lines were used in the study.

Palaeontology and Archaeology

Specimen provenance	*Provide provenance information for specimens and describe permits that were obtained for the work (including the name of the issuing authority, the date of issue, and any identifying information). Permits should encompass collection and, where applicable, export.*
Specimen deposition	*Indicate where the specimens have been deposited to permit free access by other researchers.*
Dating methods	*If new dates are provided, describe how they were obtained (e.g. collection, storage, sample pretreatment and measurement), where they were obtained (i.e. lab name), the calibration program and the protocol for quality assurance OR state that no new dates are provided.*

☐ Tick this box to confirm that the raw and calibrated dates are available in the paper or in Supplementary Information.

Ethics oversight	*Identify the organization(s) that approved or provided guidance on the study protocol, OR state that no ethical approval or guidance was required and explain why not.*

Note that full information on the approval of the study protocol must also be provided in the manuscript.

Animals and other research organisms

Policy information about studies involving animals; ARRIVE guidelines recommended for reporting animal research, and Sex and Gender in Research

Laboratory animals	6–10-week-old female immunocompromised mice; strain NRGS (NOD.Cg-Rag1tm1Momll2rgtm1Wjl); source (DKFZ breeding)
Wild animals	The study did not include wild animals
Reporting on sex	The study used only female mice. Female mice were selected for this study to allow for effective randomization into groups with comparable tumour sizes following the first positive MRI measurements. Male mice would have been less suitable for group housing due to their potential aggressive behaviour.
Field-collected samples	The study did not include field-collected samples.
Ethics oversight	Regierungspräsidium Karlsruhe, Germany

Note that full information on the approval of the study protocol must also be provided in the manuscript.

Clinical data

Policy information about clinical studies

All manuscripts should comply with the ICMJEguidelines for publication of clinical research and a completedCONSORT checklist must be included with all submissions.

Clinical trial registration	*Provide the trial registration number from ClinicalTrials.gov or an equivalent agency.*
Study protocol	*Note where the full trial protocol can be accessed OR if not available, explain why.*
Data collection	*Describe the settings and locales of data collection, noting the time periods of recruitment and data collection.*
Outcomes	*Describe how you pre-defined primary and secondary outcome measures and how you assessed these measures.*

Dual use research of concern

Policy information about dual use research of concern

Hazards

Could the accidental, deliberate or reckless misuse of agents or technologies generated in the work, or the application of information presented in the manuscript, pose a threat to:

No	Yes	
☐	☐	Public health
☐	☐	National security
☐	☐	Crops and/or livestock
☐	☐	Ecosystems
☐	☐	Any other significant area

Experiments of concern

Does the work involve any of these experiments of concern:

No	Yes	
☐	☐	Demonstrate how to render a vaccine ineffective
☐	☐	Confer resistance to therapeutically useful antibiotics or antiviral agents
☐	☐	Enhance the virulence of a pathogen or render a nonpathogen virulent
☐	☐	Increase transmissibility of a pathogen
☐	☐	Alter the host range of a pathogen
☐	☐	Enable evasion of diagnostic/detection modalities
☐	☐	Enable the weaponization of a biological agent or toxin
☐	☐	Any other potentially harmful combination of experiments and agents

Plants

Seed stocks	*Report on the source of all seed stocks or other plant material used. If applicable, state the seed stock centre and catalogue number. If plant specimens were collected from the field, describe the collection location, date and sampling procedures.*
Novel plant genotypes	*Describe the methods by which all novel plant genotypes were produced. This includes those generated by transgenic approaches, gene editing, chemical/radiation-based mutagenesis and hybridization. For transgenic lines, describe the transformation method, the number of independent lines analyzed and the generation upon which experiments were performed. For gene-edited lines, describe the editor used, the endogenous sequence targeted for editing, the targeting guide RNA sequence (if applicable) and how the editor was applied.*
Authentication	*Describe any authentication procedures for each seed stock used or novel genotype generated. Describe any experiments used to assess the effect of a mutation and, where applicable, how potential secondary effects (e.g. second site T-DNA insertions, mosiacism, off-target gene editing) were examined.*

ChIP-seq

Data deposition

☐ Confirm that both raw and final processed data have been deposited in a public database such as GEO.

☐ Confirm that you have deposited or provided access to graph files (e.g. BED files) for the called peaks.

Data access links<br>*May remain private before publication.*	*For "Initial submission" or "Revised version" documents, provide reviewer access links. For your "Final submission" document, provide a link to the deposited data.*
Files in database submission	*Provide a list of all files available in the database submission.*
Genome browser session<br>(e.g. UCSC)	*Provide a link to an anonymized genome browser session for "Initial submission" and "Revised version" documents only, to enable peer review. Write "no longer applicable" for "Final submission" documents.*

Methodology

Replicates	*Describe the experimental replicates, specifying number, type and replicate agreement.*
Sequencing depth	*Describe the sequencing depth for each experiment, providing the total number of reads, uniquely mapped reads, length of reads and whether they were paired- or single-end.*
Antibodies	*Describe the antibodies used for the ChIP-seq experiments; as applicable, provide supplier name, catalog number, clone name, and lot number.*
Peak calling parameters	*Specify the command line program and parameters used for read mapping and peak calling, including the ChIP, control and index files used.*

Data quality	*Describe the methods used to ensure data quality in full detail, including how many peaks are at FDR 5% and above 5-fold enrichment.*
Software	*Describe the software used to collect and analyze the ChIP-seq data. For custom code that has been deposited into a community repository, provide accession details.*

Flow Cytometry

Plots

Confirm that:

☐ The axis labels state the marker and fluorochrome used (e.g. CD4-FITC).

☐ The axis scales are clearly visible. Include numbers along axes only for bottom left plot of group (a 'group' is an analysis of identical markers).

☐ All plots are contour plots with outliers or pseudocolor plots.

☐ A numerical value for number of cells or percentage (with statistics) is provided.

Methodology

Sample preparation	*Describe the sample preparation, detailing the biological source of the cells and any tissue processing steps used.*
Instrument	*Identify the instrument used for data collection, specifying make and model number.*
Software	*Describe the software used to collect and analyze the flow cytometry data. For custom code that has been deposited into a community repository, provide accession details.*
Cell population abundance	*Describe the abundance of the relevant cell populations within post-sort fractions, providing details on the purity of the samples and how it was determined.*
Gating strategy	*Describe the gating strategy used for all relevant experiments, specifying the preliminary FSC/SSC gates of the starting cell population, indicating where boundaries between "positive" and "negative" staining cell populations are defined.*

☐ Tick this box to confirm that a figure exemplifying the gating strategy is provided in the Supplementary Information.

Magnetic resonance imaging

Experimental design

Design type	*Indicate task or resting state; event-related or block design.*
Design specifications	*Specify the number of blocks, trials or experimental units per session and/or subject, and specify the length of each trial or block (if trials are blocked) and interval between trials.*
Behavioral performance measures	*State number and/or type of variables recorded (e.g. correct button press, response time) and what statistics were used to establish that the subjects were performing the task as expected (e.g. mean, range, and/or standard deviation across subjects).*

Acquisition

Imaging type(s)	*Specify: functional, structural, diffusion, perfusion.*
Field strength	3T
Sequence & imaging parameters	For lesion detection, T2 weighted imaging was performed using a T2_TurboRARE sequence: TE = 48 ms, TR = 3350 ms, FOV 20x20 mm, slice thickness 1.0mm, averages = 3, Scan Time 2 min 40 s, echo spacing 12 ms, rare factor 10, slices 20, image size 192x192.
Area of acquisition	whole brain

Diffusion MRI ☐ Used ☐ Not used

Preprocessing

Preprocessing software	*Provide detail on software version and revision number and on specific parameters (model/functions, brain extraction, segmentation, smoothing kernel size, etc.).*
Normalization	*If data were normalized/standardized, describe the approach(es): specify linear or non-linear and define image types used for transformation OR indicate that data were not normalized and explain rationale for lack of normalization.*

Normalization template | *Describe the template used for normalization/transformation, specifying subject space or group standardized space (e.g. original Talairach, MNI305, ICBM152) OR indicate that the data were not normalized.*

Noise and artifact removal | *Describe your procedure(s) for artifact and structured noise removal, specifying motion parameters, tissue signals and physiological signals (heart rate, respiration).*

Volume censoring | *Define your software and/or method and criteria for volume censoring, and state the extent of such censoring.*

Statistical modeling & inference

Model type and settings | *Specify type (mass univariate, multivariate, RSA, predictive, etc.) and describe essential details of the model at the first and second levels (e.g. fixed, random or mixed effects; drift or auto-correlation).*

Effect(s) tested | *Define precise effect in terms of the task or stimulus conditions instead of psychological concepts and indicate whether ANOVA or factorial designs were used.*

Specify type of analysis: ☐ Whole brain ☐ ROI-based ☐ Both

Statistic type for inference | *Specify voxel-wise or cluster-wise and report all relevant parameters for cluster-wise methods.*

(See Eklund et al. 2016)

Correction | *Describe the type of correction and how it is obtained for multiple comparisons (e.g. FWE, FDR, permutation or Monte Carlo).*

Models & analysis

n/a	Involved in the study
☐	☐ Functional and/or effective connectivity
☐	☐ Graph analysis
☐	☐ Multivariate modeling or predictive analysis

Functional and/or effective connectivity | *Report the measures of dependence used and the model details (e.g. Pearson correlation, partial correlation, mutual information).*

Graph analysis | *Report the dependent variable and connectivity measure, specifying weighted graph or binarized graph, subject- or group-level, and the global and/or node summaries used (e.g. clustering coefficient, efficiency, etc.).*

Multivariate modeling and predictive analysis | *Specify independent variables, features extraction and dimension reduction, model, training and evaluation metrics.*
